# Pulse control protocols for preserving coherence in dipolar-coupled nuclear spin baths

**DOI:** 10.1038/s41467-019-11160-6

**Published:** 2019-07-17

**Authors:** A. M. Waeber, G. Gillard, G. Ragunathan, M. Hopkinson, P. Spencer, D. A. Ritchie, M. S. Skolnick, E. A. Chekhovich

**Affiliations:** 1 Department of Physics and Astronomy, University of Sheffield, Sheffield, S3 7RH UK; 2 Walter Schottky Institut and Physik-Department, Technische Universität München, Am Coulombwall 4, 85748 Garching, Germany; 3 Department of Electronic and Electrical Engineering, University of Sheffield, Sheffield, S1 3JD UK; 4 Cavendish Laboratory, University of Cambridge, Cambridge, CB3 0HE UK

**Keywords:** Quantum dots, Semiconductors, Quantum physics, Magnetic properties and materials

## Abstract

Coherence of solid state spin qubits is limited by decoherence and random fluctuations in the spin bath environment. Here we develop spin bath control sequences which simultaneously suppress the fluctuations arising from intrabath interactions and inhomogeneity. Experiments on neutral self-assembled quantum dots yield up to a five-fold increase in coherence of a bare nuclear spin bath. Numerical simulations agree with experiments and reveal emergent thermodynamic behaviour where fluctuations are ultimately caused by irreversible conversion of coherence into many-body quantum entanglement. Simulations show that for homogeneous spin baths our sequences are efficient with non-ideal control pulses, while inhomogeneous bath coherence is inherently limited even under ideal-pulse control, especially for strongly correlated spin-9/2 baths. These results highlight the limitations of self-assembled quantum dots and advantages of strain-free dots, where our sequences can be used to control the fluctuations of a homogeneous nuclear spin bath and potentially improve electron spin qubit coherence.

## Abstract (web summary)

Fluctuating nuclear spin ensembles are a significant decoherence mechanism for solid-state spin qubits. Here the authors introduce an approach to controlling and extending the coherence of a nuclear spin bath around self-assembled quantum dots and gain insight into the many-body dynamics.

## Introduction

The excellent spin–photon interface of confined charges in III–V semiconductor quantum dots (QDs) has recently attracted a lot of attention for potential applications in photon-mediated quantum networks^1–3^. The large optical dipole moment of QDs makes ultrafast optical spin control feasible and permits unrivalled entanglement generation rates^4–6^. On the other hand, the coherence properties of the electron or hole spin qubit are strongly affected by hyperfine interaction with the fluctuating spin bath of the ~10^5^ constituent nuclei of the QD^7,8^. Although hyperfine-induced qubit dephasing can be reduced using dynamical decoupling^9,10^, this approach requires a significant number of additional qubit manipulations and rapidly loses efficiency with increasing spin bath inhomogeneity, induced, e.g. by nuclear quadrupolar effects^11–15^.

There is a complementary pathway of controlling the nuclear spin bath itself with pulsed nuclear magnetic resonance (NMR)^16,17^. The system of a central (electron) spin qubit coupled to a nuclear spin bath is characterized by a complex hierarchy of interactions and dynamics timescales, meaning that achieving optimal control of the combined electron–nuclear spin coherence is a difficult problem. A simpler starting point is the problem of tailoring the coherence of a bare many-body nuclear spin bath (without a central spin). Examples for controlling spin–spin interactions are found in NMR spectroscopy, where sequences such as WAHUHA^18^ and MREV^19,20^ are used to average out dipolar couplings selectively. However, these solid echo sequences do not remove inhomogeneous broadening. By contrast, dynamical decoupling sequences based on *π*-pulse trains suppress inhomogeneous dephasing^21–23^, but exacerbate dipolar dephasing through the parasitic effect of instantaneous diffusion^24–26^. Thus, a different class of control sequences is required to remove both the effect of inhomogeneity and dipolar interactions.

In this work, we introduce pulse sequences which combine the features of dynamical decoupling with those of solid echoes and are designed to preserve coherence of an arbitrary quantum state of a spin bath. These combined Hahn and solid echo (CHASE) pulse sequences are tested using first-principle quantum mechanical simulations and experiments on neutral InGaAs/GaAs self-assembled QDs, where different isotopes offer access to spin baths with distinct regimes of inhomogeneous broadening and correlations in a many-body system. Even without the electron, the evolution of a bare spin bath reveals unexpected phenomena: in strongly disordered spin baths, where inhomogeneous broadening significantly exceeds the intrabath interactions, the ability to preserve coherence via global control is inherently limited—this case applies to self-assembled QDs, where spin-9/2 indium nuclei with their strong flip-flop coupling impose the ultimate limit on the maximum achievable spin bath coherence. In the case of homogeneous spin baths, we find that cyclic application of CHASE sequences can efficiently suppress spin–spin entanglement, leading to significant extension of coherence times even under nonideal control pulses. This highlights the advantages of spin qubits with homogeneous spin environments, such as dilute donor spins^26,27^, defect centres^28^, or strain-free GaAs/AlGaAs quantum dots^9,10,29^. We show that many-body decoherence emerges naturally under unitary evolution^30^: similar to the second law of thermodynamics, where useful energy is irreversibly dissipated into wasteful heat, the coherence is irreversibly converted into multipartite spin–spin entanglement. While it is not possible to eliminate spin bath dynamics completely, CHASE control sequences can transform random fluctuations into more deterministic evolution, which can in principle be decoupled from the qubit using standard control schemes^17^. More broadly, CHASE sequences may be used in quantum thermodynamic applications to preserve coherences, which play an important role in extracting work from nanoscale systems^31–33^.

## Results

### Design of CHASE pulse sequences

An intuitive approach used previously to extend nuclear spin lifetimes in silicon and diamond^27,28^ is to combine solid echo *π*/2-pulse cycles with refocusing *π*-pulses in order to suppress both the inhomogeneous dephasing and dipolar couplings. Here, we employ a rigorous average Hamiltonian theory (AHT)^34^, which is a form of perturbation theory based on Magnus expansion^35^. Using AHT as a benchmark tool (see details in Supplementary Note 1), we systematically analyse various combinations of *π*- and *π*/2-pulses to find those that maximise the spin bath coherence while minimising the pulse sequence length. The evolution of a nuclear spin bath **I**_*i*_ is analysed under a given pulse cycle in a strong external magnetic field *B*_z_. We take into account a dipolar coupling term ${\cal{H}}_{\mathrm{d}}^{{\mathrm{zz}}}$ as well as a generic resonance offset Hamiltonian ${\cal{H}}_0^{\mathrm{z}}$, which describes inhomogeneous resonance broadening due to, e.g. chemical shifts or static quadrupolar interaction

1 $$\begin{array}{*{20}{l}} {\cal{H}} \hfill & = \hfill & {{\cal{H}}_0^{\mathrm{z}} + {\cal{H}}_{\mathrm{d}}^{{\mathrm{zz}}}} \hfill \\ {} \hfill & = \hfill & {h\mathop {\sum}\limits_i {\mathrm{\Delta }} \nu _iI_{i,{\mathrm{z}}} + h\,\mathop {\sum}\limits_{i < j} {\nu _{ij}} \left( {2I_{i,{\mathrm{z}}}I_{j,{\mathrm{z}}} - I_{i,{\mathrm{x}}}I_{j,{\mathrm{x}}} - I_{i,{\mathrm{y}}}I_{j,{\mathrm{y}}}} \right),} \hfill \end{array}$$

where *ν*_*ij*_ is the dipolar coupling constant between two spins **I**_*i*_ and **I**_*j*_ and Δ*ν*_*i*_ denotes the resonance frequency offset of the *i*th nuclear spin. A larger spread in the Δ*ν*_*i*_ values describes a spin bath with a larger disorder. The free induction decay (FID) of transverse magnetisation under this Hamiltonian is described by a rate ${\mathrm{\Gamma }} \propto 1/T_2^ \ast \propto \sqrt {\langle {\mathrm{\Delta }}\nu _i^2\rangle }$.

**Fig. 1:**
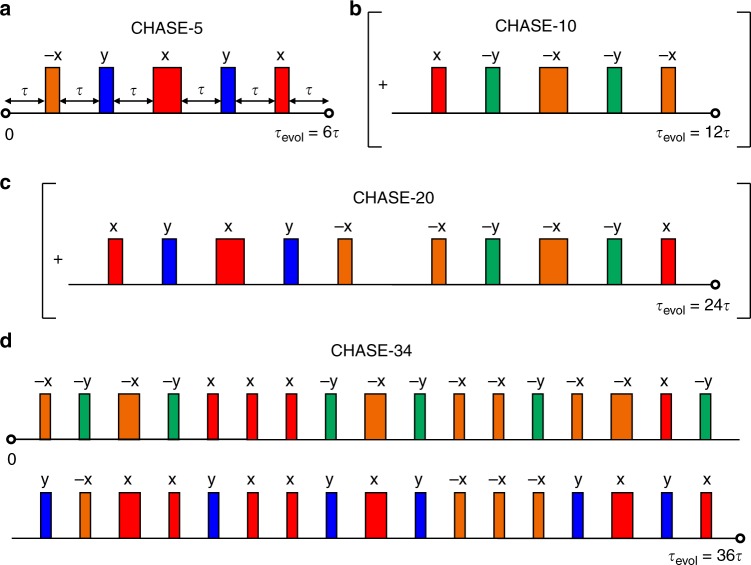
Pulse sequences for refocusing inhomogeneous and dipolar broadening. **a** The combined Hahn and solid echo sequence CHASE-5 with cycle time *t*_c_ = 3*t*_*π*_ + *τ*_evol_ consisting of the total gate time 3*t*_*π*_ and total free evolution time *τ*_evol_ = 6*τ* (where *τ* ≫ *t*_*π*_ is the free evolution time between two pulses). **b** Extension to CHASE-10, which is less sensitive to finite pulse durations *t*_*π*_ > 0. **c** Using symmetry considerations, a further optimised sequence CHASE-20 is constructed. **d** The longest sequence CHASE-34 with a total gate time 20*t*_*π*_ has the best refocusing capability for *t*_*π*_ → 0. The radio frequency (rf) carrier phases are *φ*_x_ = 0, *φ*_−x_ = *π* for ±x rotations around the $\hat e_{\mathrm{x}}$ axis and *φ*_y_ = *π*/2, *φ*_−y_ = 3*π*/2 for ±y rotations around $\hat e_{\mathrm{y}}$. Narrow pulses indicate *π*/2-rotations with pulse time *t*_*π*_/2 and broad pulses correspondingly represent *π*-rotations with duration *t*_*π*_

The shortest cycle (CHASE-5) giving a noticeable coherence increase contains only five pulses and is illustrated in Fig. 1a. Assuming infinitely short pulses (*t*_*π*_ → 0), the zeroth-order average Hamiltonian ∝ Γ vanishes. The leading residual contribution to decoherence is a first-order term ∝ ℏ*t*_c_Γ^2^ mixing contributions from the inhomogeneous broadening Hamiltonian and the dipolar interaction^36,37^:

2 $$\bar {\cal{H}}_{{\mathrm{CHASE}} - {\mathrm{5}}} = \frac{{{\mathrm{i}}t_{\mathrm{c}}}}{{18\hbar }}[{\cal{H}}_{\mathrm{d}}^{{\mathrm{zz}}} - {\cal{H}}_{\mathrm{d}}^{{\mathrm{xx}}},{\cal{H}}_0^{\mathrm{y}}] + {\cal{O}}(\hbar t_{\mathrm{c}}^2{\mathrm{\Gamma }}^3),$$

where *t*_c_ is the full cycle time and ${\cal{H}}_{\mathrm{d}}^{{\mathrm{xx}}}$, ${\cal{H}}_0^{\mathrm{y}}$ are the dipolar and inhomogeneous broadening Hamiltonians acting along orthogonal equatorial axes $\hat e_{\mathrm{x}}$ and $\hat e_{\mathrm{y}}$.

Under realistic experimental conditions, the *t*_*π*_ → 0 assumption is often not justified. For finite pulse durations *t*_*π*_, the zeroth-order average Hamiltonian does not vanish under CHASE-5. However, we can obtain an average Hamiltonian of the form of Eq. (2) even for finite *t*_*π*_ by extending the cycle to CHASE-10 (Fig. 1b). Furthermore, by adding the pulse block shown in Fig. 1c, we can symmetrise the cycle to CHASE-20 and remove the first-order mixing term, condensing the average Hamiltonian to $\bar {\cal{H}}_{{\mathrm{CHASE}} - {\mathrm{20}}} \propto {\cal{O}}(\hbar t_{\mathrm{c}}^2{\mathrm{\Gamma }}^3)$ independent of the pulse duration *t*_*π*_. Finally, we identify the CHASE-34 sequence (Fig. 1d), which reduces the average Hamiltonian to a second-order mixing term for *t*_*π*_ → 0 but has non-vanishing lower-order terms for finite pulse durations. A comprehensive overview of the AHT calculations and residual Hamiltonians can be found in Supplementary Note 1.

### CHASE NMR experiments on self-assembled quantum dots

We study the performance of these sequences experimentally on individual charge-free InGaAs QDs with ~10^5^ nuclear spins. The average fractions of the isotopes in a dot are^11^: 50% (^75^As), ~24% (^69^Ga), ~16% (^71^Ga), ~9.5% (^115^In) and <0.5% (^113^In). We follow the ODNMR pump–probe scheme used in ref. ^38^: the QD sample is kept at low temperature (*T* = 4.2 K) and is subjected to a strong magnetic field *B*_z_ = 8 T. Using a confocal microscopy setup in Faraday configuration, we prepare the nuclear spin bath optically through polarisation-selective pumping of an exciton transition (dynamic nuclear polarisation). In this way, we achieve hyperfine-mediated spin bath polarisation degrees of up to 65%^39,40^. Radio frequency (rf) fields are coupled to the QD via a multi-winding copper coil in close proximity to the sample. Changes in the final bath polarisation are probed with a weak optical pulse measuring the energy splitting of the neutral exciton Zeeman doublet^11^.

We perform resonant pulsed NMR measurements on the inhomogeneously broadened central spin transition −1/2 $\leftrightarrow$ +1/2 of the spin-3/2 ^75^As (inhomogeneous width of $\Delta \nu _{{\mathrm{inh}}} \sim 40\,{\mathrm{kHz}}$), spin-3/2 $^{71}$Ga $({\mathrm{\Delta }}\nu _{{\mathrm{inh}}} \sim 10\,{\mathrm{kHz}})$ and spin-9/2 ^115^In $(\Delta \nu _{{\mathrm{inh}}} \sim 30\,{\mathrm{kHz}})$ nuclear ensembles^11,38^. In each experiment, the population difference of the *I*_z_ = ±1/2 states is enhanced via adiabatic rapid passage^38,41^ and only one isotope is manipulated with rf pulses, while the spins in the |*I*_z_| ≥ 3/2 states as well as other isotopes are left to evolve freely during the pulse sequence. The phases of the *π*-pulses in all sequences are chosen to produce spin rotations around the $\hat e_{\mathrm{x}}$ axis of the rotating frame. In each experiment, a *π*/2-pulse is applied prior to the multipulse cycle to initialise the transverse spin polarisation by converting energy into coherence^31^. We conduct experiments with initial *π*/2-rotation around the $\hat e_{\mathrm{x}}$ axis (Carr–Purcell or CP-like sequences^42^, denoted as −X) and around the $\hat e_{\mathrm{y}}$ axis (Carr–Purcell–Meiboom–Gill or CPMG-like sequences^43^, −Y): in this way, we distinguish between a genuine improvement of the spin coherence and spin locking effects^44–46^, which only stabilise spin magnetisation along a certain direction. A final *π*/2-pulse is an inverse of the initialisation pulse and projects the spin echo polarisation onto the $\hat e_{\mathrm{z}}$ axis for optical readout^38^.

**Fig. 2:**
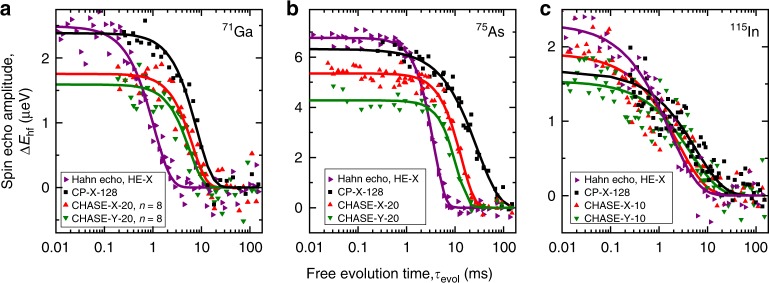
Nuclear spin echoes in a single neutral InGaAs QD. **a** Decay of the ^71^Ga NMR spin echo measured via hyperfine shift Δ*E*_hf_ as a function of the total free evolution time *τ*_evol_ under different control sequences. Symbols mark experimental data and solid lines show best fits with Eq. (3). **b**, **c** Results for ^75^As and ^115^In, respectively

**Fig. 3:**
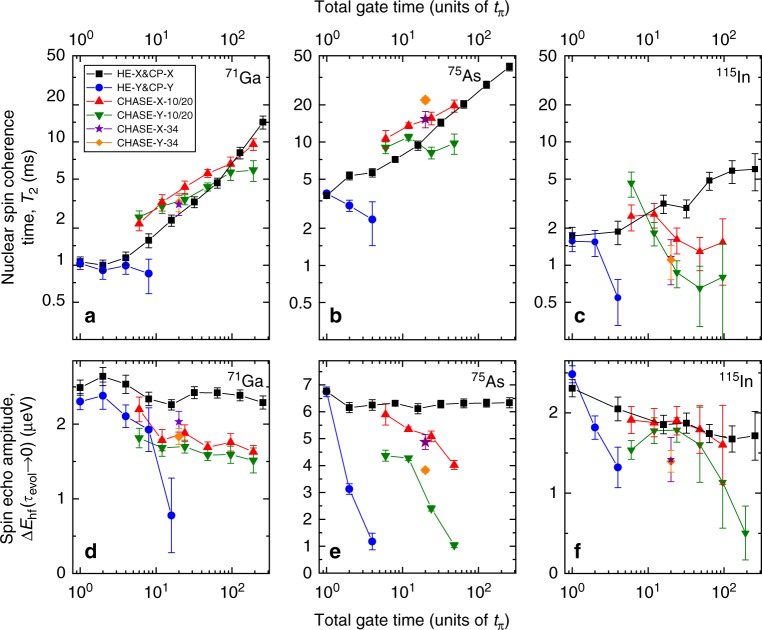
Experimental nuclear spin decoherence in an InGaAs quantum dot. **a**–**c** Dependence of the fitted ^71^Ga (**a**), ^75^As (**b**) and ^115^In (**c**) spin coherence time *T*_2_ on the number of sequence cycles *n* expressed as total rf gate time (in units of *t*_*π*_). Results are shown for Hahn echo, CP and CHASE pulse sequences. **d**–**f** Respective fitted spin echo amplitude Δ*E*_hf_(*τ*_evol_ → 0) at short free evolution time *τ*_evol_ → 0 as a function of the total gate time. Error bars mark 90% confidence intervals. The data for one cycle of Hahn echo (HE) and CHASE-10 are combined with the data for integer cycle numbers *n* of CP and CHASE-20, respectively. The *π*-pulse durations used in these experiments are *t*_*π*_ = 4.3 μs (^71^Ga), *t*_*π*_ = 5.7 μs (^75^As) and *t*_*π*_ = 4.7 μs (^115^In)

Figure 2 shows representative experimental dependencies of the spin echo amplitude on the total free evolution time *τ*_evol_ over *n* sequence cycles for ^71^Ga (a), ^75^As (b) and ^115^In (c) nuclei. The decay of the echo measured in terms of hyperfine shift Δ*E*_hf_ is modeled by a compressed exponential decay function

3 $${\mathrm{\Delta }}E_{{\mathrm{hf}}}(\tau _{{\mathrm{evol}}}) = {\mathrm{\Delta }}E_{{\mathrm{hf}}}(\tau _{{\mathrm{evol}}} \to 0) \cdot {\mathrm{e}}^{ - (\tau _{{\mathrm{evol}}}/T_2)^\beta },$$

where *β* is a compression factor^47^, *T*_2_ describes decoherence of the spin bath during free evolution, while reduction of the echo amplitude Δ*E*_hf_(*τ*_evol_ → 0) at short free evolution compared with the initial spin polarisation Δ*E*_hf_(*t* = 0) quantifies the imperfections of pulse spin rotations. The fitted values of *T*_2_ and Δ*E*_hf_(*τ*_evol_ → 0) are plotted in Fig. 3a–f for the three studied isotopes at different *n* expressed in terms of the total control gate time in units of *t*_*π*_. The compression factor *β* depends on the spectral properties of the random process responsible for the decoherence^24^. From fitting, we find *β*≈1.0–2.0 for ^71^Ga and ^75^As, agreeing with the values typically found for various spin systems, whereas *β*≈0.7–1.0 in the case of ^115^In due to the non-exponential echo decay.

### Control of nuclear spin coherence in quantum dots

We start by analysing the common behaviour of all the isotopes under CP-X and CP-Y sequences with an alternating pulse carrier phase (sequence cycle −*τ*/2 − *π*_x_ − *τ* − *π*_−x_ − *τ*/2−). For increasing *n*, the echo amplitude is robust and *T*_2_ increases under CP-X (black squares in Fig. 3a–f), whereas under CP-Y (blue circles), the echo amplitude is rapidly reduced, owing to the limited available rf power resulting in deviation from the ideal hard pulses (see Supplementary Note 3). The contrasting behaviour of *T*_2_ under alternating phase CP-X/Y has been observed in other systems^44–46,48^ and has been attributed variably to spin locking^48,49^ or stimulated echoes^50^. Here, we ascribe the increase of *T*_2_ under CP-X to a form of pulsed spin locking arising from dipolar evolution during the finite-duration *π*-pulses^49^: our interpretation is based on the observation that the spin lock disappears for small pulse-to-cycle time ratios *t*_*π*_/*t*_c_ (see Supplementary Note 2).

We now examine the spin bath coherence under CHASE-10/20 and CHASE-34. Figure 3a–c shows an increase of *T*_2_ under CHASE, compared with the Hahn echo *T*_2_ for all isotopes. This increase occurs both under −X and −Y initialisation signifying a genuine increase of nuclear spin coherence, as opposed to pulsed spin locking under CP-X. However, the effect of CHASE sequence cycling (increasing *n*, and correspondingly increasing the total rf gate time) depends strongly on the isotope. For ^71^Ga, there is a steady increase in *T*_2_ up to $T_2^{{\mathrm{CHASE}} - {\mathrm{Y}} - {\mathrm{20}}} \approx 6.0\,{\mathrm{ms}}$ at *n* = 16 (compared with Hahn echo $T_2^{{\mathrm{HE}} - {\mathrm{X}}/{\mathrm{Y}}} \approx 1.0\,{\mathrm{ms}}$, Fig. 3a), revealing the expected convergence of the average Hamiltonian to zero with reducing cycle time *t*_c_. The growth of *T*_2_ with increasing *n* is accompanied by a gradual reduction of the echo amplitude (Fig. 3d)—the result of the nonideal (finite-duration) control pulses. By contrast, *T*_2_ is nearly constant for ^75^As (Fig. 3b), and the echo amplitude reduction is more pronounced, owing to the larger inhomogeneous broadening Δ*ν*_*i*_ and limited rf pulse amplitude. While there is no difference for ^71^Ga, in the case of ^75^As, CHASE-34 somewhat improves coherence over CHASE-20, showing that with growing inhomogeneous broadening, higher-order Hamiltonian terms have a stronger impact on decoherence than the evolution during nonideal control pulses.

A rather different picture is observed for the spin-9/2 ^115^In: beyond one cycle of CHASE-10, sequence cycling actually reduces *T*_2_ (Fig. 3c). Notably, this is not related to imperfect spin rotations, as reduction in echo amplitudes (Fig. 3f) is less pronounced than for ^75^As (Fig. 3f), suggesting a fundamentally different mechanism.

### First-principle numerical simulations

**Fig. 4:**
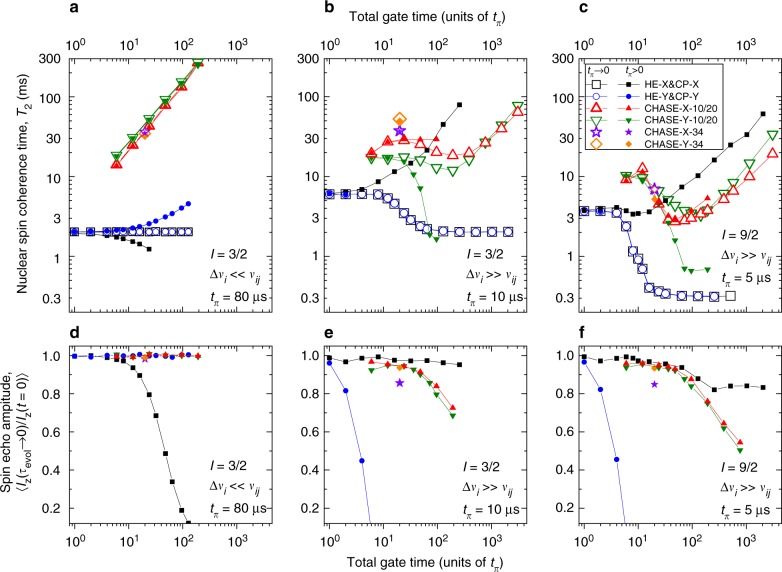
Simulated decoherence of a dipolar-coupled nuclear spin ensemble. Numerically simulated spin echo decay under different pulse sequences (see Supplementary Note 4 for representative data and further details) is fitted using Eq. (3) to derive nuclear spin coherence time *T*_2_ and the normalised spin echo amplitude 〈*I*_z_(*τ*_evol_ → 0)/*I*_z_(*t* = 0)〉 for short free evolution. **a**–**c** Fitted *T*_2_ as a function of the total gate time. **d**–**f** Corresponding gate time dependence of the spin echo amplitude. The results are presented for spin-3/2 ^75^As nuclei with small inhomogeneous (quadrupolar) broadening Δ*ν*_*i*_ (**a**, **d**), ^75^As with large Δ*ν*_*i*_ (**b**, **e**) and spin-9/2 ^115^In nuclei with large Δ*ν*_*i*_ (**c**, **f**). Simulations are done for both infinitely short (*t*_*π*_ → 0, open symbols) and finite pulses (solid symbols, *t*_*π*_ = 5, 10 or 80 μs)

In order to get a better insight into the underlying mechanisms of the many-body spin decoherence, we conduct first-principle quantum mechanical simulations of the nuclear spin bath evolution. We consider an ensemble of 12 dipolar-coupled nuclei with spin *I* and study the evolution of the *I*_z_ = ±1/2 subspace under rf driving of the central transition −1/2↔ +1/2 in the limits of large (Δ*ν*_*i*_ ≫ *ν*_*ij*_) and vanishing (Δ*ν*_*i*_ ≪ *ν*_*ij*_) inhomogeneous resonance broadening. Figure 4 shows the fitted coherence times (a, b, c) and echo amplitudes (d, e, f) for simulated spin echo decay curves in the following cases: *I* = 3/2, Δ*ν*_*i*_ ≪ *ν*_*ij*_ (a, d); *I* = 3/2, Δ*ν*_*i*_ ≫ *ν*_*ij*_ (b, e); *I* = 9/2, Δ*ν*_*i*_ ≫ *ν*_*ij*_ (c, f).

The simulations with Δ*ν*_*i*_ ≫ *ν*_*ij*_ and realistic rf pulse durations (solid symbols) are in very good agreement with the experiments on QDs (Fig. 3). Namely, *T*_2_ increases under CP-X (spin locking) and the echo amplitude reduces under CP-Y with growing *n*. The absolute simulated *T*_2_ values are only a factor of ~2 larger than in the experiment (which is due to the limited number of spins in the model, see Supplementary Fig. 5). Under CHASE-10/20, there is a reduction in *T*_2_ with increasing *n* for *I* = 9/2 spins similar to the ^115^In experiment (cf. Figs. 3c and 4c); there is a strong reduction of the echo amplitude for spin-3/2 nuclei with Δ*ν*_*i*_ ≫ *ν*_*ij*_, in agreement with the ^75^As experiment (cf. Figs. 3b, e and 4b, e). Moreover, the pronounced increase of *T*_2_ for ^71^Ga with its smaller inhomogeneous broadening matches well the simulation with Δ*ν*_*i*_ ≪ *ν*_*ij*_ (cf. Figs. 3a and 4a).

Good agreement with experiments means the simulations are valid and can be used to explore the experimentally inaccessible regimes of ideal (infinitely short *t*_*π*_ → 0) control pulses (open symbols in Fig. 4). We readily find that even with perfect spin rotations (*t*_*π*_ → 0), the *T*_2_ of an inhomogeneously broadened (Δ*ν*_*i*_ ≫ *ν*_*ij*_) spin ensemble under CHASE-10/20 shows a flat region for *I* = 3/2 (Fig. 4b) and even an initial decrease with growing *n* for *I* = 9/2 (Fig. 4c), which is contrary to lim_*n*→∞_*T*_2_ = ∞, expected for the average Hamiltonian converging to zero. This unexpected discrepancy can be understood by noting that the Hahn echo (*n* = 1/2) coherence $T_2^{{\mathrm{HE}} - {\mathrm{X}}/{\mathrm{Y}}}$ of spin-3/2 nuclei is ~3 times longer for Δ*ν*_*i*_ ≫ *ν*_*ij*_ due to the freezing of the dipolar spin–spin flip-flops by inhomogeneous broadening^38^. When *n* is increased, the CP-X/Y coherence time $T_2^{{\mathrm{CP}} - {\mathrm{X}}/{\mathrm{Y}}}$ decreases (open squares and circles in Fig. 4b) and asymptotically approaches the $T_2^{{\mathrm{HE}} - {\mathrm{X}}/{\mathrm{Y}}}$ obtained for Δ*ν*_*i*_ ≪ *ν*_*ij*_ where spin flip-flops are allowed (Fig. 4a). This suggests that the fast spin rotations induced by the high repetition rate train of infinitely short *π*-pulses effectively reduce the spin lifetime, broaden the homogeneous NMR linewidths and re-enable the frozen dipolar flip-flops in an inhomogeneous spin bath. Further simulations show that restoration of the flip-flops at larger inhomogeneous broadening requires a proportionally larger pulse repetition rate, confirming our interpretation. The ‘heating’ of the spins by frequent control pulses is then also responsible for slow convergence of the average Hamiltonian under CHASE sequences: the growth in *T*_2_ can only be achieved when the flip-flops are fully ‘thawed’ (at total gate times ≳200*t*_*π*_ in Fig. 4b, c). When projected to the *I*_z_ = ±1/2 states, the dipolar Hamiltonian has the form

4 $${\cal{H}}_{\mathrm{d}}^{{\mathrm{zz}}} \propto 2I_{i,{\mathrm{z}}}I_{j,{\mathrm{z}}} - (I + 1/2)^2(I_{i,{\mathrm{x}}}I_{j,{\mathrm{x}}} + I_{i,{\mathrm{y}}}I_{j,{\mathrm{y}}}),$$

so the role of the flip-flops is inherently most pronounced for *I* = 9/2 nuclei explaining the particularly strong reduction of ^115^In coherence under long control sequences. Flip-flops in the *I*_z_ = ±1/2 subspace are possible only for pairs with opposite spins. Thus, the heating of spins by control pulses will depend on the actual spin state. In other words, under frequent spin rotations, the many-body Hamiltonian itself becomes state-dependent, invalidating the AHT approximation. This mechanism is the main practical limitation in controlling the coherence of a disordered many-body spin system via global spin rotations.

### Emergent thermodynamic behaviour in a spin bath

**Fig. 5:**
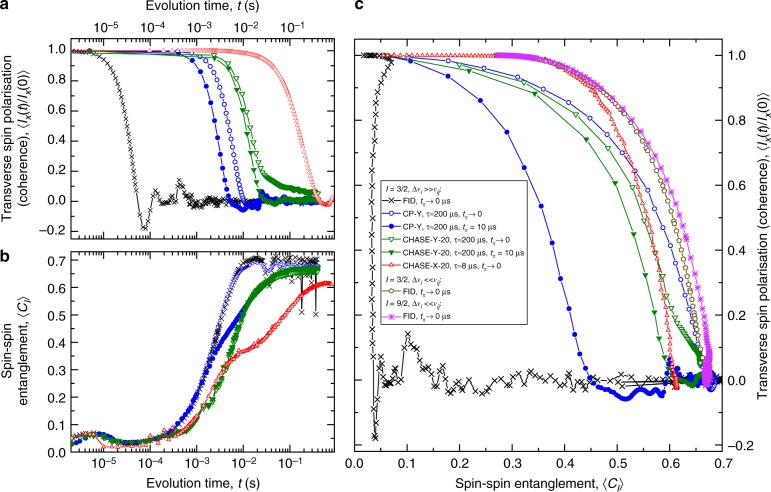
Simulated evolution of spin coherence and entanglement of a dipolar-coupled nuclear spin bath. Simulations are for the *I*_z_ = ±1/2 spin subspace under ideal (*t*_*π*_ → 0, open symbols) and finite (*t*_*π*_ = 10 μs, solid symbols) control pulses. **a** Time evolution of the average transverse magnetisation 〈*I*_x_(*t*)〉 normalised by the initial magnetisation 〈*I*_x_(0)〉 for spin-3/2 nuclei with large inhomogeneous broadening Δ*ν*_*i*_ ≫ *ν*_*ij*_. Simulations are for free induction decay (FID, initialisation *π*/2-pulse only) and several cyclic pulse sequences. In the case of the cyclic sequences, 〈*I*_x_(*t*)〉 is plotted only at the times of spin echoes. Note that the results here are simulated with a constant pulse-to-pulse delay *τ*, whereas the data in Fig. 2a–c are measured with a constant number *n* of pulse sequence repeats. **b** Evolution of the averaged spin–spin entanglement 〈*C*_*I*_〉 for the same simulations as in (**a**). **c** Trajectories in the 〈*C*_*I*_〉 − 〈*I*_x_〉 phase space for the results of (**a**) and (**b**) as well as additional trajectories for small inhomogeneity (Δ*ν*_*i*_ ≪ *ν*_*ij*_) and spin-9/2 nuclei

In addition to the measurable transverse spin magnetisation (coherence), numerical simulations permit probing quantities that are not directly observed in experiment. In particular, we now examine the evolution of the multipartite spin–spin quantum entanglement and demonstrate its fundamental role in the decoherence of an interacting spin bath. We quantify entanglement using the basis-independent intrinsic coherence measure *C*_*I*_ introduced in ref. ^51^. Figure 5a shows the simulated time evolution of the transverse spin magnetisation 〈*I*_x_(*t*)〉 of spin-3/2 nuclei with large inhomogeneous broadening Δ*ν*_*i*_ ≫ *ν*_*ij*_. The results are averaged over randomly chosen initial eigenstates, and calculations are done for a fixed free evolution time *τ* between pulses. Figure 5b shows the evolution of the entanglement measure 〈*C*_*I*_(*t*)〉 for the same simulations, whereas Fig. 5c presents the same results and additional data for a homogeneous bath Δ*ν*_*i*_ ≪ *ν*_*ij*_ and spin-9/2 nuclei as trajectories in the entanglement-coherence 〈*C*_*I*_〉 − 〈*I*_x_〉 phase space.

Under free induction decay (FID, crosses in Fig. 5), coherence 〈*I*_x_(*t*)〉 is lost rapidly within ~30 μs, before the onset of entanglement 〈*C*_*I*_〉, which happens within ~3 ms (with some dips arising from the quasiperiodic evolution of a small spin ensemble). Application of the CP-Y sequence with ideal pulses (*t*_*π*_ → 0, open circles) has no effect on entanglement rate but extends coherence decay to the same timescale as entanglement. Going from CP-Y to CHASE-20 (open triangles), we observe a slowdown both in the 〈*I*_x_〉 decay and 〈*C*_*I*_〉 growth, with the actual timescales strongly dependent on *τ*. The systematic relation between coherence and entanglement is demonstrated in Fig. 5c. Firstly, we find that evolution of the spin ensemble is always accompanied by a nearly monotonic conversion of coherence into entanglement. Secondly, we observe that despite different decoherence and entanglement rates, CP-Y and CHASE-20 with ideal pulses (*t*_*π*_ → 0) result in similar 〈*C*_*I*_〉 − 〈*I*_x_〉 trajectories—extensive simulations for CP-X/Y and CHASE-X/Y show that this similarity holds for a wide range of pulse-to-pulse delays *τ* and applies to the case of a small inhomogeneity Δ*ν*_*i*_ ≪ *ν*_*ij*_ as well as spin-9/2 nuclei (a limited selection is shown in Fig. 5c to keep the graph readable). This gives an empirical evidence for the existence of a universal optimal trajectory, where 〈*I*_x_〉 has maximal possible value at any given 〈*C*_*I*_〉. Figure 5c reveals the factors leading to suboptimal trajectories: in the absence of pulsed spin control (FID), transverse coherence in an inhomogeneous ensemble (Δ*ν*_*i*_ ≫ *ν*_*ij*_) is rapidly lost well before emergence of any significant spin–spin entanglement. Furthermore, in an inhomogeneous system under pulsed spin control, going from ideal (*t*_*π*_ → 0, open symbols) to finite (*t*_*π*_ = 10 μs, solid symbols) control pulses, the trajectories become non-ideal with a shift towards the FID trajectory—this is very pronounced in the case of CP-Y (circles), while for CHASE-Y-20 with the same *τ*≈200 μs, the trajectory is closer to ideal, demonstrating the robustness of the CHASE sequence.

The evolution of coherence and entanglement can be interpreted in the framework of thermodynamics, i.e. without the need to know the exact form of the internal interaction Hamiltonian. The simulation results presented above show that transverse magnetisation 〈*I*_x_〉 (coherence) of the spin system is analogous to work, i.e. a useful resource—in the case of nuclear spins, this work can actually be extracted, e.g. in the form of a current induced in a coil. By contrast, the entanglement 〈*C*_*I*_〉 is analogous to a wasteful heat, which cannot be used to generate coherence or extract work. The true decoherence of the spin ensemble is then associated with the irreversible generation of entanglement 〈*C*_*I*_〉 via the spin–spin interactions^52,53^. For example, the apparent quick loss of coherence in the FID of an inhomogeneous ensemble (Δ*ν*_*i*_ ≫ *ν*_*ij*_) does not involve entanglement and in fact can be reversed by applying a *π*-pulse train (CP sequence). By contrast, once the maximum level of entanglement is reached (〈*C*_*I*_〉≈ 0.67 in Fig. 5c), it is impossible to recover coherence, regardless of the pulse sequence applied. It is important to note that there are no artificial sources of decoherence in the presented simulations, implying the evolution is unitary^54^ and thus demonstrating that the irreversible loss of coherence due to entanglement emerges naturally^30^ in a system of just a few dipolar-coupled spins. Entanglement due to the nonideal (finite) control pulses can be understood, since these pulses are equivalent to Hamiltonian quenches, which are generally known to induce irreversible evolution^30^. The difficulty of improving *I* = 9/2 indium spin coherence with pulsed control (Figs. 3c and 4c) also finds its explanation within the quantum thermodynamics framework, since stronger spin–spin correlations, which scale as ∝ (*I* + 1/2)^2^, generally result in faster entanglement growth^53^ and more irreversible evolution^55^.

## Discussion

Based on our results, we conclude with the following interpretation of the relation between disorder, coherence and entanglement in dipolar-coupled spin baths. Suppression of spin–spin entanglement growth is required to extend spin coherence. This can be achieved by applying CHASE pulse cycles which converge the spin–spin interaction Hamiltonian to zero with reducing cycle time *t*_c_ → 0. Realistic (finite-duration) control pulses impose a lower limit on *t*_c_. Despite that, the Hamiltonian in a homogeneous spin bath does converge under CHASE, predicting at least a factor of ~100 growth in coherence time under realistic parameters (Fig. 4a). A robust performance under nonideal soft pulses is a unique feature of CHASE distinguishing it from the previously introduced time-suspension sequences^28,56–58^ (see additional simulations in Supplementary Note 5). In a strongly inhomogeneous (disordered) spin bath, the spin–spin interactions are difficult to eliminate, even if ideal control pulses were possible (Fig. 4b, c), as control pulses themselves make the spin–spin interactions state-dependent, disrupting the convergence of the Hamiltonian. Finite control pulses impose further restrictions, limiting the maximum coherence time that can be realistically achieved in an inhomogeneous spin bath.

In the case of bare nuclear spin baths, CHASE can be used, for example, to preserve the quantum information transferred to the nuclei from the electron spin^59^. Another potential application is for dynamical suppression of the nuclear spin bath decoherence and fluctuations, which limit the electron spin qubit coherence in quantum dots. In order for dynamical suppression to be efficient, a significant fraction of the bath nuclei must be controlled. In self-assembled (InGaAs) quantum dots, the high-spin states |*I*_z_| > 1/2 cannot be manipulated with coherent rf pulses, due to the extreme (few-megahertz) resonance broadening. While this can be overcome by nuclear spin hyperpolarisation^29^ and a subsequent population transfer^38,41^ to the |*I*_z_| = 1/2 states, the real fundamental limitation for the InGaAs dots arises from the strongly correlated nature of the indium spin-9/2 subsystem. A more promising area of application are lattice-matched GaAs/AlGaAs quantum dots (both electrostatic^15^ and epitaxial^29^) or II–VI quantum dots^60^, where quadrupolar inhomogeneous broadening is small or absent, enabling significant extension of coherence times and the ability to operate in a wide range of magnetic fields ≳1–10 mT, requiring only that the nuclear Zeeman splitting is larger than the quadrupolar and dipolar nuclear spin interactions.

Interaction with a central (electron) spin complicates the coherent dynamics compared with the bare nuclear spin bath case. For example, the nuclear spin echo coherence in InGaAs electron-charged dots is few tens of microseconds^41^, but is expected to be longer in GaAs dots, due to the larger number of nuclei and smaller hyperfine interaction per nucleus. Using few microsecond-long rf pulses, it should be possible to examine extension of nuclear spin coherence under CHASE sequences in the presence of an electron. This would be a basis for exploring CHASE control of the nuclei, synchronised with electron spin qubit control, with the aim of creating a deterministically evolving nuclear spin environment and leading to extended electron spin qubit coherence—this approach can compliment existing techniques, such as preparation of the nuclear spin bath in a narrowed state via electron–nuclear feedback^61,62^.

More broadly, CHASE is promising for dopants and point defects in diamond, silicon and silicon carbide, where it can be applied both to the nuclear spin bath and directly to electron spin qubits to enhance their coherence beyond the limits of standard dynamical decoupling protocols by suppressing the instantaneous spin diffusion^16,26^ arising from electron–electron dipolar interactions. Further optimisation of spin bath decoherence freezing can be explored using techniques such as optimal control^63^.

## Supplementary information

Supplementary Information

## Data Availability

The data that support the findings of this study are available from the corresponding authors upon reasonable request.
