# Supplementary material for: Pulse control protocols for preserving coherence in dipolar-coupled nuclear spin baths
Source: Nat Commun. 2019 Jul 17;10:3157. doi: 10.1038/s41467-019-11160-6 (PMC6637143; doi:10.1038/s41467-019-11160-6)
Supplement: Supplementary file 1 — Supplementary Information [file 41467_2019_11160_MOESM1_ESM.pdf]

Supplementary Information: Pulse control protocols for preserving coherence in dipolar-coupled nuclear spin baths

A. M. Waeber,* G. Gillard, G. Ragunathan, M. Hopkinson, P. Spencer, D. A. Ritchie, M. S. Skolnick, and E. A. Chekhovich†

Supplementary Note 1. DESIGN OF PULSE SEQUENCES USING AVERAGE HAMILTONIAN THEORY

Average Hamiltonian theory (AHT) is an established tool for the theoretical characterisation and analysis of pulse sequences for magnetic resonance spin control[1–3]. Within certain constraints it allows the time evolution of a given spin Hamiltonian under interaction with a periodic time-dependent external magnetic field to be approximated. We use AHT to determine how well a frequency offset Hamiltonian $\mathcal{H}_0^{\rm z}$ (arising from inhomogeneous resonance broadening) and a dipolar coupling term $\mathcal{H}_{\rm d}^{\rm zz}$ can be suppressed simultaneously by the CHASE sequences introduced in the main text.

To this end, we consider a nuclear spin ensemble $\mathbf{I}_i$ with spin 1/2. The evolution of the wavefunction $\psi(t)$ describing the state of the nuclear spin bath is determined by the Schrödinger equation:

$$\partial\psi(t)/\partial t = -({\rm i}/\hbar)\mathcal{H}(t)\psi(t) \ , \tag{1}$$

$$\mathcal{H}(t) = \mathcal{H}_{\rm L}^{\rm z} + \mathcal{H}_0^{\rm z} + \mathcal{H}_{\rm d}^{\rm zz} + \mathcal{H}_{\rm rf}(t) \ , \tag{2}$$

where the Hamiltonian $\mathcal{H}(t)$ is the sum of the Larmor term $\hat{H}_{\rm L}$ describing interaction of the spins with a static magnetic field $B_{\rm z}$ along the $\hat{e}_{\rm z}$ axis, the offset term $\mathcal{H}_0^{\rm z}$ describing static resonance frequency shifts, the dipolar term $\mathcal{H}_{\rm d}^{\rm zz}$ describing nuclear-nuclear spin interaction and the radio-frequency (rf) term $\mathcal{H}_{\rm rf}(t)$ describing the effect of the oscillating magnetic field inducing nuclear magnetic resonance.

We use transformation into the frame rotating around the direction of the static magnetic field ($\hat{e}_{\rm z}$ axis) at the radio-frequency. In this way the effect of the static magnetic field is eliminated ($\mathcal{H}_{\rm L}^{\rm z} = 0$) and the oscillating rf field becomes static (see Section 5.5 in Ref.[4]). The explicit time dependence in $\mathcal{H}_{\rm rf}(t)$ is then only due to the pulsed nature of the rf field.

The individual terms are explicitly defined as

$$\mathcal{H}_0^{\rm z} = h\sum_i \Delta\nu_i I_{i,{\rm z}} \ , \tag{3}$$

$$\mathcal{H}_{\rm d}^{\rm zz} = h\sum_{i<j} \nu_{ij}\left(3I_{i,{\rm z}}I_{j,{\rm z}} - \mathbf{I}_i\cdot\mathbf{I}_j\right) \ , \tag{4}$$

$$\mathcal{H}_{\rm rf}(t) = -h\nu_{\rm rf}(t)\sum_i I_{i,\varphi} \ . \tag{5}$$

In the studied quantum dots (QDs) the resonance offset term $\mathcal{H}_0^{\rm z}$ is dominated by the inhomogeneous static quadrupolar frequency shifts ($\Delta\nu_i$ for the i-th nuclear spin), although in other systems $\Delta\nu_i$ could also include different static frequency offsets such as chemical shifts or magnetic field

gradients. In addition, we consider a truncated dipolar coupling term $\mathcal{H}_{\mathrm{d}}^{\mathrm{zz}}$ with coupling strength

$$\nu_{ij} = \frac{\mu_0}{4\pi}\frac{\hbar}{2\pi}\frac{\gamma^2}{2}\frac{1-3\cos^2\theta}{r^3} \ , \tag{6}$$

between two spins $\mathbf{I}_i$ and $\mathbf{I}_j$. Here, $\mu_0 = 4\pi \cdot 10^{-7}$ N A^{-2} is the magnetic constant, γ is the nuclear gyromagnetic ratio, and r denotes the length of the vector connecting the two spins, which forms angle θ with the $\hat{e}_{\mathrm{z}}$ axis.

Interaction with resonant rf pulses is described by the time-dependent term $\mathcal{H}_{\mathrm{rf}}(t)$ where the field amplitude $\nu_{\mathrm{rf}}(t) = \nu_0$ during a pulse and $\nu_{\mathrm{rf}}(t) = 0$ otherwise. The spin operator I_φ determines the in-plane rotation axis about which the spin bath precesses under the rf field with a given phase φ:

$$I_\varphi = I_{\mathrm{x}}\cos\varphi + I_{\mathrm{y}}\sin\varphi \ . \tag{7}$$

Here, we want to study the spin bath evolution under the internal static terms $\mathcal{H}_{\mathrm{int}} = \mathcal{H}_0^{\mathrm{z}} + \mathcal{H}_{\mathrm{d}}^{\mathrm{zz}}$ in the interaction frame of $\mathcal{H}_{\mathrm{rf}}(t)$. AHT can give an approximate description of the time evolution after one or more full rf cycles if three conditions are met: (i) The rf Hamiltonian is periodic over the cycle time t_{c}, i.e. $\mathcal{H}_{\mathrm{rf}}(t+t_{\mathrm{c}}) = \mathcal{H}_{\mathrm{rf}}(t)$. (ii) The net spin rotation after a full rf cycle is a multiple of 2π. (iii) Since AHT is a perturbation method in terms of t_{c}/T_2^*, the solution only converges quickly if $|\mathcal{H}_0^{\mathrm{z}}|t_{\mathrm{c}}/\hbar \ll 1$ and $|\mathcal{H}_{\mathrm{d}}^{\mathrm{zz}}|t_{\mathrm{c}}/\hbar \ll 1$.

We write the total time-evolution operator as

$$\mathcal{U}(t) = \mathcal{T}\exp\left[-\frac{\mathrm{i}}{\hbar}\int_0^t dt'\mathcal{H}(t')\right] = \mathcal{U}_{\mathrm{rf}}(t)\mathcal{U}_{\mathrm{int}}(t) \ , \tag{8}$$

with Dyson time-ordering operator $\mathcal{T}$ and

$$\mathcal{U}_{\mathrm{rf}}(t) = \mathcal{T}\exp\left[-\frac{\mathrm{i}}{\hbar}\int_0^t dt'\mathcal{H}_{\mathrm{rf}}(t')\right] \ , \tag{9}$$

$$\mathcal{U}_{\mathrm{int}}(t) = \mathcal{T}\exp\left[-\frac{\mathrm{i}}{\hbar}\int_0^t dt'\tilde{\mathcal{H}}_{\mathrm{int}}(t')\right] \ , \tag{10}$$

where we introduced the toggling frame Hamiltonian

$$\tilde{\mathcal{H}}_{\mathrm{int}}(t) = \mathcal{U}_{\mathrm{rf}}^{-1}(t)\mathcal{H}_{\mathrm{int}}\mathcal{U}_{\mathrm{rf}}(t) \ . \tag{11}$$

While an exact solution for Eq. (10) is generally challenging to find, an approximate description of the spin bath evolution at times $t = n \cdot t_{\mathrm{c}}$ can be found if conditions (i)-(iii) are fulfilled. In this case, we can apply a Magnus expansion to replace the expression of Eq. (10) by an effective average Hamiltonian $\bar{\mathcal{H}}$ such that

$$\begin{aligned}\mathcal{U}_{\mathrm{int}}(nt_{\mathrm{c}}) &= \exp\left[-\frac{\mathrm{i}}{\hbar}nt_{\mathrm{c}}\bar{\mathcal{H}}\right] \\ &= \exp\left[-\frac{\mathrm{i}}{\hbar}nt_{\mathrm{c}}\left(\bar{\mathcal{H}}^{(0)} + \bar{\mathcal{H}}^{(1)} + \ldots\right)\right] \ ,\end{aligned} \tag{12}$$

with leading order terms

$$\bar{\mathcal{H}}^{(0)} = \frac{1}{t_\mathrm{c}} \int_0^{t_\mathrm{c}} \tilde{\mathcal{H}}(t)\mathrm{d}t \ , \tag{13}$$

$$\bar{\mathcal{H}}^{(1)} = \frac{-\mathrm{i}}{2t_\mathrm{c}\hbar} \int_0^{t_\mathrm{c}} \mathrm{d}t_2 \int_0^{t_2} \mathrm{d}t_1 \left[\tilde{\mathcal{H}}(t_2), \tilde{\mathcal{H}}(t_1)\right] \ , \tag{14}$$

$$\begin{aligned} \bar{\mathcal{H}}^{(2)} = \frac{1}{6t_\mathrm{c}\hbar^2} \int_0^{t_\mathrm{c}} \mathrm{d}t_3 \int_0^{t_3} \mathrm{d}t_2 \int_0^{t_2} \mathrm{d}t_1 \\ \left(\left[\tilde{\mathcal{H}}(t_1), \left[\tilde{\mathcal{H}}(t_2), \tilde{\mathcal{H}}(t_3)\right]\right] \right. \\ \left. + \left[\tilde{\mathcal{H}}(t_3), \left[\tilde{\mathcal{H}}(t_2), \tilde{\mathcal{H}}(t_1)\right]\right] \right) \ . \end{aligned} \tag{15}$$

The contributions of higher order AHT terms to $\bar{\mathcal{H}}$ scale as $t_\mathrm{c}^k \Gamma^{k+1}$ for $\bar{\mathcal{H}}^{(k)}$, with free coherence decay rate $\Gamma \propto 1/T_2^* \propto \sqrt{\langle \Delta\nu_i^2 \rangle}$. We thus see that in the limit of $n \to \infty$ cycles and cycle time $t_\mathrm{c} \to 0$, only the zeroth order term $\bar{\mathcal{H}}^{(0)}$ remains. However, in practice, higher order contributions are rarely negligible, making e.g. longer solid echo sequences such as MREV[5,6] and BR-24[3] more efficient than the shorter WAHUHA cycle[7] in many applications.

We calculate the average Hamiltonian for a spin bath interacting with a given pulse sequence as described by equations (2)-(5) using Wolfram Mathematica software with the freely-available non-commutative algebra package NCAlgebra[8]. For the zeroth order average Hamiltonian, we consider finite pulse durations where t_π is the time required for a π-rotation. In this case, the cycle time t_c is given by the sum of pulse times and pulse-to-pulse delays τ. First and second order terms are only calculated in the limit of infinitely short rf pulses $t_\pi \to 0$.

The AHT terms we obtain for the CHASE sequences presented in the main text are listed in Supplementary Table 1. For clarity, we split the k-th order average Hamiltonian into contributions from the resonance offset ($\bar{\mathcal{H}}_0^{(k)}$) and dipolar Hamiltonian ($\bar{\mathcal{H}}_\mathrm{d}^{(k)}$). For higher orders $k \geq 1$, we also include mixed terms ($\bar{\mathcal{H}}_\mathrm{d0}^{(k)}$). The full k-th order average Hamiltonian is thus given by

$$\bar{\mathcal{H}}^{(k)} = \bar{\mathcal{H}}_0^{(k)} + \bar{\mathcal{H}}_\mathrm{d}^{(k)} + \bar{\mathcal{H}}_\mathrm{d0}^{(k)} \ . \tag{16}$$

Only non-vanishing terms are listed, i.e. terms which do not appear in Supplementary Table 1 do not contribute to the total average Hamiltonian.

The definition of the additional resonance offset and dipolar coupling Hamiltonians used in Supplementary Table 1 is based on equations (3) and (4), i.e.

$$\mathcal{H}_0^\mathrm{x} = h \sum_i \Delta\nu_i I_{i,\mathrm{x}} \ , \qquad \mathcal{H}_0^\mathrm{y} = h \sum_i \Delta\nu_i I_{i,\mathrm{y}} \ , \tag{17}$$

$$\mathcal{H}_\mathrm{d}^\mathrm{xx} = h \sum_{i<j} \nu_{ij} \left(3 I_{i,\mathrm{x}} I_{j,\mathrm{x}} - \mathbf{I}_i \cdot \mathbf{I}_j\right) \ , \qquad \mathcal{H}_\mathrm{d}^\mathrm{yy} = h \sum_{i<j} \nu_{ij} \left(3 I_{i,\mathrm{y}} I_{j,\mathrm{y}} - \mathbf{I}_i \cdot \mathbf{I}_j\right) \ . \tag{18}$$

Supplementary Table 1. Overview of AHT terms up to second order calculated for the CHASE sequences presented in the main text. The cycle of each sequence is given in curly brackets where x, y ($-$x, $-$y) represent positive (negative) $\pi/2$ rotations around the respective axes, x^2, y^2, $-$x^2, $-$y^2 stand for the π rotations, while τ and 2τ are the free evolution intervals. Zeroth order terms are calculated assuming finite pulse duration t_π whereas $t_\pi \to 0$ is assumed for higher order terms. Unlisted terms $\bar{\mathcal{H}}_0^{(0),(1),(2)}$, $\bar{\mathcal{H}}_{\rm d}^{(0),(1),(2)}$, $\bar{\mathcal{H}}_{\rm d0}^{(1),(2)}$ are zero.

AHT term	Sequence
	CHASE-5
	$\{\tau,\text{-x},\tau,\text{y},\tau,\text{x}^2,\tau,\text{y},\tau,\text{x},\tau\}$
$\bar{\mathcal{H}}_0^{(0)}$	$\frac{2t_\pi}{\pi t_{\rm c}}\mathcal{H}_0^{\rm z}$
$\bar{\mathcal{H}}_{\rm d}^{(0)}$	$\frac{{\rm i}t_\pi}{\pi t_c}\left([\mathcal{H}_{\rm d}^{\rm xx}, I_{\rm z}] + [\mathcal{H}_{\rm d}^{\rm yy}, I_{\rm x} - I_{\rm z}] - [\mathcal{H}_{\rm d}^{\rm zz}, I_{\rm x}]\right)$
$\bar{\mathcal{H}}_{\rm d0}^{(1)}$	$\frac{{\rm i}t_{\rm c}}{18\hbar}[\mathcal{H}_{\rm d}^{\rm zz} - \mathcal{H}_{\rm d}^{\rm xx}, \mathcal{H}_0^{\rm y}]$
$\bar{\mathcal{H}}_{\rm d}^{(2)}$	$\frac{1}{2}\left(\frac{t_{\rm c}}{18\hbar}\right)^2[\mathcal{H}_{\rm d}^{\rm zz} - \mathcal{H}_{\rm d}^{\rm xx}, [\mathcal{H}_{\rm d}^{\rm zz}, \mathcal{H}_{\rm d}^{\rm yy}]]$
$\bar{\mathcal{H}}_{\rm d0}^{(2)}$	$3\left(\frac{t_{\rm c}}{18\hbar}\right)^2\left([\mathcal{H}_0^{\rm y}, [\mathcal{H}_0^{\rm y}, \mathcal{H}_{\rm d}^{\rm xx} - \mathcal{H}_{\rm d}^{\rm zz}]] + [\mathcal{H}_{\rm d}^{\rm zz} - \mathcal{H}_{\rm d}^{\rm yy}, [\mathcal{H}_0^{\rm z}, \mathcal{H}_0^{\rm y}]]\right)$
	CHASE-10
	$\{\tau,\text{-x},\tau,\text{y},\tau,\text{x}^2,\tau,\text{y},\tau,\text{x},2\tau,\text{x},\tau,\text{-y},\tau,\text{-x}^2,\tau,\text{-y},\tau,\text{-x},\tau\}$
$\bar{\mathcal{H}}_{\rm d0}^{(1)}$	$\frac{{\rm i}t_{\rm c}}{36\hbar}[\mathcal{H}_{\rm d}^{\rm zz} - \mathcal{H}_{\rm d}^{\rm xx}, \mathcal{H}_0^{\rm y}]$
$\bar{\mathcal{H}}_{\rm d}^{(2)}$	$\frac{1}{2}\left(\frac{t_{\rm c}}{36\hbar}\right)^2[\mathcal{H}_{\rm d}^{\rm zz} - \mathcal{H}_{\rm d}^{\rm xx}, [\mathcal{H}_{\rm d}^{\rm zz}, \mathcal{H}_{\rm d}^{\rm yy}]]$
$\bar{\mathcal{H}}_{\rm d0}^{(2)}$	$3\left(\frac{t_{\rm c}}{36\hbar}\right)^2[\mathcal{H}_0^{\rm y}, [\mathcal{H}_0^{\rm y}, \mathcal{H}_{\rm d}^{\rm xx} - \mathcal{H}_{\rm d}^{\rm zz}]]$
	CHASE-20
	$\{\tau,\text{-x},\tau,\text{y},\tau,\text{x}^2,\tau,\text{y},\tau,\text{x},2\tau,\text{x},\tau,\text{-y},\tau,\text{-x}^2,\tau,\text{-y},\tau,\text{-x},2\tau,\text{x},\tau,\text{y},\tau,\text{x}^2,\tau,\text{y},\tau,\text{-x},2\tau,\text{-x},\tau,\text{-y},\tau,\text{-x}^2,\tau,\text{-y},\tau,\text{x},\tau\}$
$\bar{\mathcal{H}}_{\rm d}^{(2)}$	$\frac{1}{2}\left(\frac{t_{\rm c}}{72\hbar}\right)^2[\mathcal{H}_{\rm d}^{\rm zz} - \mathcal{H}_{\rm d}^{\rm xx}, [\mathcal{H}_{\rm d}^{\rm zz}, \mathcal{H}_{\rm d}^{\rm yy}]]$
$\bar{\mathcal{H}}_{\rm d0}^{(2)}$	$3\left(\frac{t_{\rm c}}{72\hbar}\right)^2[\mathcal{H}_0^{\rm y}, [\mathcal{H}_0^{\rm y}, \mathcal{H}_{\rm d}^{\rm xx} - \mathcal{H}_{\rm d}^{\rm zz}]]$
	CHASE-34
	$\{\tau,\text{-x},\tau,\text{-y},\tau,\text{-x}^2,\tau,\text{-y},\tau,\text{x},\tau,\text{x},\tau,\text{x},\tau,\text{-y},\tau,\text{-x}^2,\tau,\text{-y},\tau,\text{-x},\tau,\text{-x},\tau,\text{-y},\tau,\text{-x},\tau,\text{-x}^2,\tau,\text{x},\tau,\text{-y},2\tau,$ $\text{y},\tau,\text{-x},\tau,\text{x}^2,\tau,\text{x},\tau,\text{y},\tau,\text{x},\tau,\text{x},\tau,\text{y},\tau,\text{x}^2,\tau,\text{y},\tau,\text{-x},\tau,\text{-x},\tau,\text{-x},\tau,\text{y},\tau,\text{x}^2,\tau,\text{y},\tau,\text{x},\tau\}$
$\bar{\mathcal{H}}_0^{(0)}$	$\frac{4t_\pi}{\pi t_{\rm c}}(\mathcal{H}_0^{\rm x} + \mathcal{H}_0^{\rm y} - \mathcal{H}_0^{\rm z})$
$\bar{\mathcal{H}}_{\rm d}^{(0)}$	$\frac{{\rm i}t_\pi}{\pi t_c}\left(4[\mathcal{H}_{\rm d}^{\rm xx}, I_{\rm y} - I_{\rm z}] + 2[\mathcal{H}_{\rm d}^{\rm yy}, I_{\rm x} + 2I_{\rm z}] - 2[\mathcal{H}_{\rm d}^{\rm zz}, I_{\rm x} + 2I_{\rm y}] + i\pi\mathcal{H}_{\rm d}^{\rm zz}\right)$
$\bar{\mathcal{H}}_{\rm d0}^{(2)}$	$3\left(\frac{t_{\rm c}}{108\hbar}\right)^2\left(\frac{2}{3}[\mathcal{H}_0^{\rm y}, [\mathcal{H}_0^{\rm y}, \mathcal{H}_{\rm d}^{\rm xx} - \mathcal{H}_{\rm d}^{\rm zz}]] + \frac{2}{3}[\mathcal{H}_{\rm d}^{\rm zz} - \mathcal{H}_{\rm d}^{\rm yy}, [\mathcal{H}_0^{\rm z}, \mathcal{H}_0^{\rm y}]] - \frac{1}{3}[\mathcal{H}_{\rm d}^{\rm zz} - \mathcal{H}_{\rm d}^{\rm xx}, [\mathcal{H}_0^{\rm z}, \mathcal{H}_0^{\rm x}]]\right)$

As discussed in the main text, we see that CHASE-5 has a non-vanishing zeroth order contribution if the pulse duration t_π is non-negligible. In order to suppress spin bath dynamics using cycles of CHASE-5, it is therefore crucial to minimise the ratio $t_\pi/t_{\rm c}$. The subsequent longer sequences are insensitive to finite pulse durations in zeroth order and leave progressively fewer higher order AHT terms in the $t_\pi \to 0$ limit. CHASE-34 forms an exception to this behaviour. While most efficient

in suppressing the spin dynamics under ideal conditions, this cycle is also prone to finite-duration pulse effects as the zeroth order resonance offset ($\bar{\mathcal{H}}_0^{(0)}$) and dipolar ($\bar{\mathcal{H}}_\mathrm{d}^{(0)}$) average Hamiltonians contribute to dephasing under realistic experimental conditions. We note that the $\bar{\mathcal{H}}_0^{(0)}$ term can be eliminated if all six π rotations have the same sense. However, the CHASE-34 sequence with three negative and three positive π rotations shown in Table 1 has a better bandwidth (see Supplementary Note 3) and in experiments on quantum dot nuclei shows an overall better coherence preservation performance.

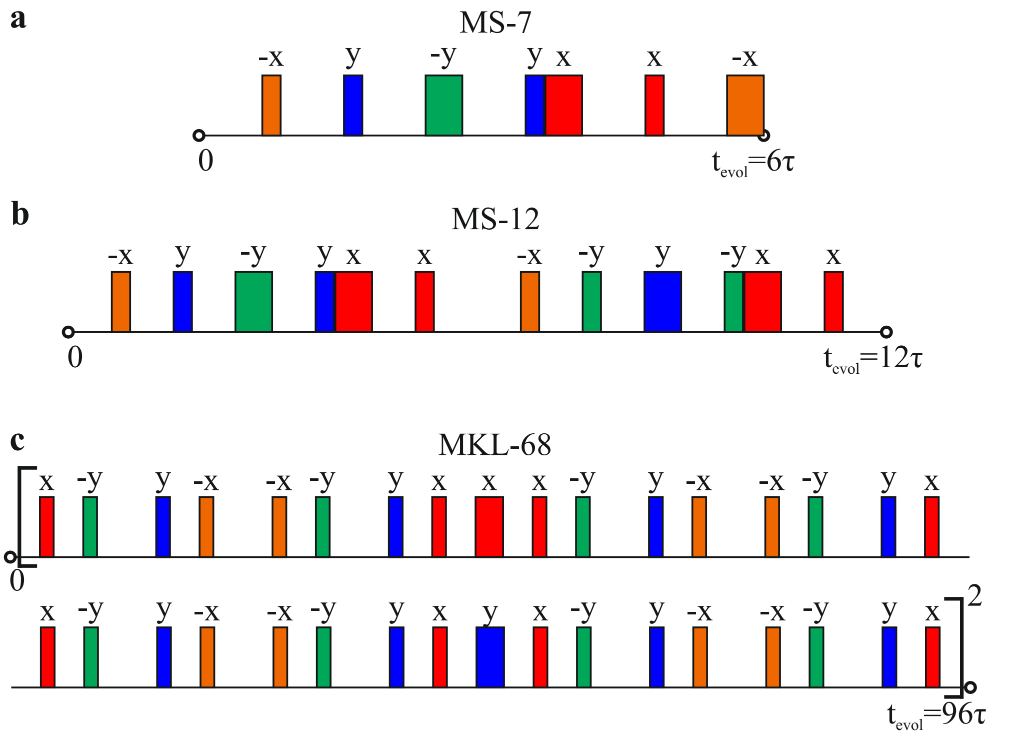

Supplementary Figure 1. **Previously introduced pulse sequences for refocusing inhomogeneous and dipolar broadening.** **a** MS-7 cycle as introduced by Moiseev and Skrebnev in Ref[9]. **b** MS-12 cycle as introduced by Moiseev and Skrebnev in Ref[10]. **c** MKL-68 cycle as described and used by Maurer, Kucsko et al. in Ref[11]. Labels are defined as in Fig. 1 of the main text.

For comparison, we also calculate the AHT terms of alternative sequences from literature which have been proposed or used with the aim of suppressing both dipolar coupling and frequency offset terms. These results are listed separately in Supplementary Table 2.

The MS-7 pulse cycle[9] (Supplementary Fig. 1b) yields average Hamiltonians identical to those of CHASE-5 in the short pulse limit. Its extension[10] to MS-12 (Supplementary Fig. 1c) removes odd-order AHT terms owing to its symmetry properties. However, unlike the longer CHASE sequences it is not robust against decoherence in case of a finite pulse duration t_π.

Supplementary Table 2. Overview of AHT terms calculated for other cycles referred to in the main text.

AHT term	MS-7[9]
$\bar{\mathcal{H}}_0^{(0)}$	$\frac{2t_\pi}{\pi t_c}\left(\mathcal{H}_0^{\mathrm{y}}+\mathcal{H}_0^{\mathrm{z}}\right)$
$\bar{\mathcal{H}}_{\mathrm{d}}^{(0)}$	$\frac{\mathrm{i}t_\pi}{\pi t_c}\left([\mathcal{H}_{\mathrm{d}}^{\mathrm{yy}}-\mathcal{H}_{\mathrm{d}}^{\mathrm{zz}},I_{\mathrm{x}}]+\frac{5}{2}\mathrm{i}\pi\mathcal{H}_{\mathrm{d}}^{\mathrm{xx}}+\mathrm{i}\pi\mathcal{H}_{\mathrm{d}}^{\mathrm{yy}}+2\mathrm{i}\pi\mathcal{H}_{\mathrm{d}}^{\mathrm{zz}}\right)$
$\bar{\mathcal{H}}_{\mathrm{d0}}^{(1)}$	$\frac{\mathrm{i}t_c}{18\hbar}[\mathcal{H}_{\mathrm{d}}^{\mathrm{zz}}-\mathcal{H}_{\mathrm{d}}^{\mathrm{xx}},\mathcal{H}_0^{\mathrm{y}}]$
$\bar{\mathcal{H}}_{\mathrm{d}}^{(2)}$	$\frac{1}{2}\left(\frac{t_c}{18\hbar}\right)^2[\mathcal{H}_{\mathrm{d}}^{\mathrm{zz}}-\mathcal{H}_{\mathrm{d}}^{\mathrm{xx}},[\mathcal{H}_{\mathrm{d}}^{\mathrm{zz}},\mathcal{H}_{\mathrm{d}}^{\mathrm{yy}}]]$
$\bar{\mathcal{H}}_{\mathrm{d0}}^{(2)}$	$3\left(\frac{t_c}{18\hbar}\right)^2\left([\mathcal{H}_0^{\mathrm{y}},[\mathcal{H}_0^{\mathrm{y}},\mathcal{H}_{\mathrm{d}}^{\mathrm{xx}}-\mathcal{H}_{\mathrm{d}}^{\mathrm{zz}}]]+[\mathcal{H}_{\mathrm{d}}^{\mathrm{zz}}-\mathcal{H}_{\mathrm{d}}^{\mathrm{yy}},[\mathcal{H}_0^{\mathrm{z}},\mathcal{H}_0^{\mathrm{y}}]]\right)$
	MS-12[10]
$\bar{\mathcal{H}}_{\mathrm{d}}^{(0)}$	$\frac{\mathrm{i}t_\pi}{\pi t_c}\left([\mathcal{H}_{\mathrm{d}}^{\mathrm{yy}}-\mathcal{H}_{\mathrm{d}}^{\mathrm{zz}},2I_{\mathrm{x}}]+\frac{5}{2}\mathrm{i}\pi\mathcal{H}_{\mathrm{d}}^{\mathrm{xx}}+\frac{1}{2}\mathrm{i}\pi\mathcal{H}_{\mathrm{d}}^{\mathrm{yy}}+\frac{5}{2}\mathrm{i}\pi\mathcal{H}_{\mathrm{d}}^{\mathrm{zz}}\right)$
$\bar{\mathcal{H}}_{\mathrm{d}}^{(2)}$	$\frac{1}{2}\left(\frac{t_c}{36\hbar}\right)^2[\mathcal{H}_{\mathrm{d}}^{\mathrm{zz}}-\mathcal{H}_{\mathrm{d}}^{\mathrm{xx}},[\mathcal{H}_{\mathrm{d}}^{\mathrm{zz}},\mathcal{H}_{\mathrm{d}}^{\mathrm{yy}}]]$
$\bar{\mathcal{H}}_{\mathrm{d0}}^{(2)}$	$3\left(\frac{t_c}{36\hbar}\right)^2\left([\mathcal{H}_0^{\mathrm{y}},[\mathcal{H}_0^{\mathrm{y}},\mathcal{H}_{\mathrm{d}}^{\mathrm{xx}}-\mathcal{H}_{\mathrm{d}}^{\mathrm{zz}}]]+[\mathcal{H}_{\mathrm{d}}^{\mathrm{zz}}-\mathcal{H}_{\mathrm{d}}^{\mathrm{yy}},[\mathcal{H}_0^{\mathrm{z}},\mathcal{H}_0^{\mathrm{y}}]]\right)$
	MKL-68[11]
$\bar{\mathcal{H}}_{\mathrm{d}}^{(0)}$	$\frac{t_\pi}{t_c}\left(-2\mathcal{H}_{\mathrm{d}}^{\mathrm{xx}}+6\mathcal{H}_{\mathrm{d}}^{\mathrm{yy}}-\mathcal{H}_{\mathrm{d}}^{\mathrm{zz}}\right)$
$\bar{\mathcal{H}}_{\mathrm{d}}^{(2)}$	$\frac{1}{2}\left(\frac{t_c}{288\hbar}\right)^2[\mathcal{H}_{\mathrm{d}}^{\mathrm{zz}}-\mathcal{H}_{\mathrm{d}}^{\mathrm{xx}},[\mathcal{H}_{\mathrm{d}}^{\mathrm{zz}},\mathcal{H}_{\mathrm{d}}^{\mathrm{yy}}]]$
$\bar{\mathcal{H}}_{\mathrm{d0}}^{(2)}$	$-3\left(\frac{t_c}{288\hbar}\right)^2[\mathcal{H}_0^{\mathrm{y}},[\mathcal{H}_0^{\mathrm{y}},\mathcal{H}_{\mathrm{d}}^{\mathrm{xx}}-\mathcal{H}_{\mathrm{d}}^{\mathrm{zz}}]]$

The intuitive approach of alternating MREV cycles with π-pulses (MKL-68, Supplementary Fig. 1d) was employed by Maurer, Kucsko et al. to extend nuclear spin coherence times in diamond[11]. Again, the performance of the cycle is limited under experimental conditions by a non-vanishing zeroth-order term $\bar{\mathcal{H}}_{\mathrm{d}}^{(0)}$.

Supplementary Note 2. PULSED SPIN LOCKING

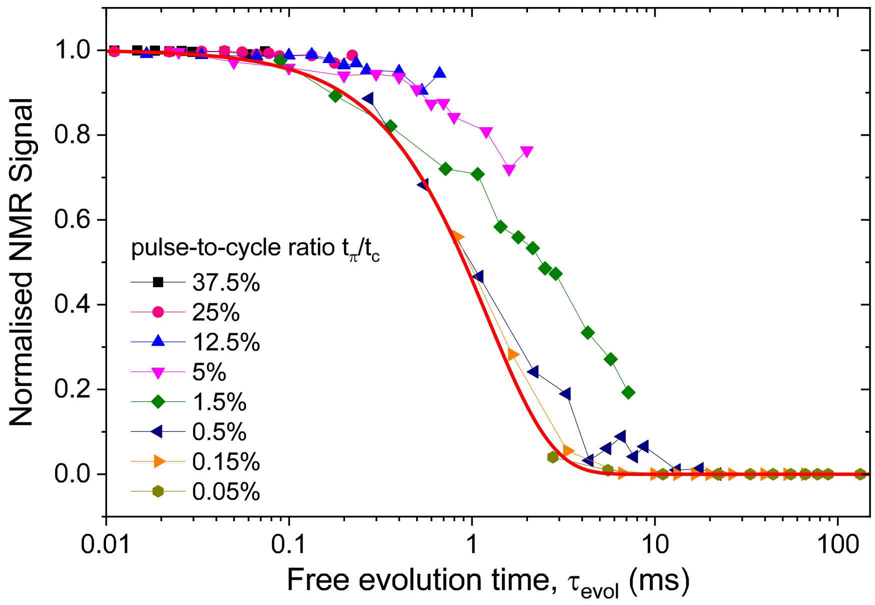

Supplementary Figure 2. **Observation of spin locking arising from spin evolution during finite control pulses.** Dependence of the experimentally measured normalised ^{71}Ga nuclear spin echo amplitude on the free evolution time τ_{evol} under the phase-alternating CP-X series tested in the main text. Each trace shows data for a fixed pulse-to-cycle ratio t_π/t_{c} and varying π-pulse number. Values are extracted from exponential decay fits to experimental data as shown in Fig. 2b of the main text. The Hahn echo decay fit is shown as a solid red line for comparison.

The extended nuclear spin polarisation decay times we observe in Fig. 3a-c of the main text under phase-alternated Carr-Purcell sequences (CP-X) are attributed to a form of pulsed spin locking described theoretically by Li et al.[12] This spin locking mechanism arises due to dipolar evolution during the non-negligible π-pulse duration t_π. As shown by the authors, in this limit application of average Hamiltonian theory yields

$$\bar{\mathcal{H}}^{(0)} = \frac{4t_\pi}{\pi t_{\mathrm{c}}}\mathcal{H}_0^{\mathrm{y}} + \frac{1}{t_{\mathrm{c}}}\left(4\tau\mathcal{H}_{\mathrm{d}}^{\mathrm{zz}} - t_\pi\mathcal{H}_{\mathrm{d}}^{\mathrm{xx}}\right) , \tag{19}$$

for a cycle $(-\tau - \pi_{\mathrm{x}} - 2\tau - \pi_{-\mathrm{x}} - \tau-)$. The static term $\propto \mathcal{H}_0^{\mathrm{y}}$ in equation (19) is subsequently removed by transformation into a second toggling frame where we time-average over a Rabi cycle in its effective field. The twice averaged Hamiltonian is

$$\bar{\bar{\mathcal{H}}}^{(0)} = -\frac{1}{t_{\mathrm{c}}}\left(2\tau - \frac{t_\pi}{2}\right)\mathcal{H}_{\mathrm{d}}^{\mathrm{yy}} . \tag{20}$$

As the initial $(\pi/2)_{\mathrm{x}}$ pulse of the CP-X sequence prepares the spin bath in the state I_{y} which commutes with $\mathcal{H}_{\mathrm{d}}^{\mathrm{yy}}$, the magnetisation is preserved or 'locked' and no spin echo decay is predicted

in zeroth order. Li et al. also examined the cases of fixed-phase CP-X as well as CP-Y with and without phase-alternation and found that, in agreement with our experimental results, no such effect is predicted for the CP-Y sequence tested in the current work[12].

Alternative mechanisms leading to prolonged coherence times under CP-X have been suggested by other authors[13,14]. However, we can confidently link our experimental observation to the pulsed spin locking mechanism outlined above. A key assumption in the transition to the second toggling frame is that the spin bath evolves slowly under the Hamiltonian $\left(4\tau\mathcal{H}_{\rm d}^{\rm zz} - t_\pi\mathcal{H}_{\rm d}^{\rm xx}\right)/t_{\rm c}$ over the relevant timescale set by the Rabi frequency $\Omega = \frac{4t_\pi\Delta\nu_i}{\pi t_{\rm c}}$ (c.f. condition (iii) for applicability of AHT in Supplementary Note 1). Hence we expect a strong dependence of the spin locking efficiency on the pulse-to-cycle time ratio $t_\pi/t_{\rm c} \in [0, 0.5]$, where $t_\pi/t_{\rm c} \to 0$ in the limit of infinitely short pulses and $t_\pi/t_{\rm c} \to 0.5$ in the limit of continuous rf excitation.

In Fig. 2a-c of the main text we show the nuclear spin polarisation decay as a function of the free evolution time for a fixed number of pulses and varying pulse spacings τ. In order to verify the dependence of spin locking on the pulse-to-cycle ratio, we now replot this experimental data as a function of the π-pulse number for different fixed pulse-to-cycle ratios. Supplementary Fig. 2 shows that the spin locking efficiency is noticeably reduced for $t_\pi/t_{\rm c} \lesssim 10\%$ and the Hahn echo decay (red solid line) is fully restored for $t_\pi/t_{\rm c} \lesssim 0.5\%$.

Supplementary Note 3. PERFORMANCE BEYOND THE HARD PULSE LIMIT

The central transitions (CTs) of the half-integer quadrupolar nuclear spins in a self-assembled QD are typically inhomogeneously broadened to $\Delta\nu_{\text{inh}} \sim 10-40$ kHz by the strain induced electric field gradients[15]. In order to apply pulse sequences uniformly to the CTs of all the nuclei, we need to ensure that rf pulses are sufficiently broadband ('hard'). In other words the pulse amplitude should be large enough to perform the desired $\pi/2$- or π-rotation even for spins I in the tails of the inhomogeneously broadened transition spectrum where the rf excitation can have a resonance offset $\Delta\nu_i \gtrsim 10$ kHz. It is often assumed implicitly that this hard pulse condition is fulfilled.

However, while this is readily achievable for small pulse numbers (for a single pulse we require $T_{\text{Rabi}} \lesssim 2/\Delta\nu_{\text{inh}} \approx 25$ μs), the hard pulse condition is increasingly difficult to meet for longer sequences. We consider the Bloch equations of motion in the rotating frame of a static magnetic field B_{z}

$$\partial\mathbf{M}(t)/\partial t = \mathbf{\Omega} \times \mathbf{M} - \mathbf{\Gamma} \cdot (\mathbf{M} - \mathbf{M}_0) \; , \tag{21}$$

describing the evolution of magnetisation $\mathbf{M} = \sum_i \gamma \mathbf{I}_i$ under an angular velocity vector $\mathbf{\Omega} = (\Omega_{\text{rf}} \cos\varphi, \Omega_{\text{rf}} \sin\varphi, 2\pi\Delta\nu_i)^{\text{T}}$ and with relaxation rate $\mathbf{\Gamma} = (T_2^{-1}, T_2^{-1}, T_1^{-1})^{\text{T}}$ and equilibrium magnetisation $\mathbf{M}_0$. Here, $\Omega_{\text{rf}} = 2\pi/T_{\text{Rabi}}$ is the resonant Rabi frequency. We see that even a small resonance offset $\Delta\nu_i$ results in a tilt of $\mathbf{\Omega}$ towards the $\hat{e}_{\text{z}}$ axis. As a consequence, an rf pulse of duration T_{Rabi} and carrier phase φ will no longer result in a perfect 2π-rotation of the magnetisation $\mathbf{M}$ about $\mathbf{\Omega}$. Instead, a small rotation angle error is introduced, which can rapidly lead to non-negligible effects as the errors of subsequent pulses add up. In practice, this is reflected in a loss of NMR signal amplitude after a pulse sequence even in the limit of short free evolution as the contribution of spins with large $\Delta\nu_i$ to the measured final ensemble magnetisation is reduced.

We consider this effect numerically for the ^{71}Ga and ^{75}As nuclear spin bath ensembles studied in this work. To quantify the 'hardness' (frequency bandwidth) over which a given pulse sequence is stable against resonance offsets, we calculate the evolution of a magnetisation vector $\mathbf{M}$ under the sequence including initialisation and readout $\pi/2$-pulses as a function of $\Delta\nu$. In order to keep our results as general as possible, we rewrite the resonance offset in terms of the inverse resonant Rabi period as $\delta = \Delta\nu T_{\text{Rabi}}$. We can then describe the resulting rotation axis as $\hat{e}_\Omega = (1+\delta^2)^{-1/2}(\cos\varphi, \sin\varphi, -\delta)^{\text{T}}$. For simplicity, our model does not consider any spin relaxation or dephasing (e.g. through dipolar interaction) and we set $T_1 = T_2 = \infty$.

Supplementary Fig. 3a,b shows the simulated dependence of the CP-X/Y sequences with alter-

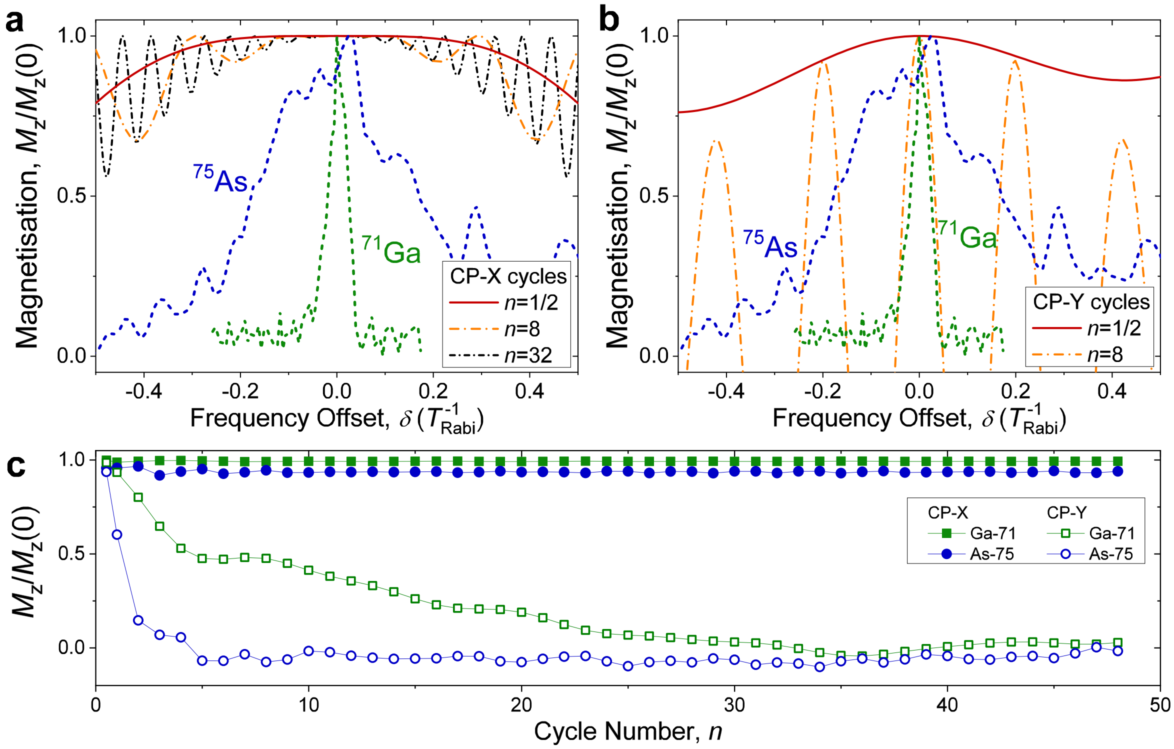

Supplementary Figure 3. **Analysis of the broadband refocusing performance of the Carr-Purcell pulse sequences. a,b** Normalised magnetisation along the $\hat{e}_z$ axis after evolution under a series of non-resonant rf pulses as a function of the resonance frequency offset δ for (a) CP-X and (b) CP-Y sequences with alternating carrier phase. Solid curves show simulated data for different cycle numbers n. Dotted lines show normalised NMR spectra of the ^{75}As (blue) and ^{71}Ga (green) central transitions with frequency axes rescaled by T_{Rabi}. **c** Weighted average of the normalised magnetisation over the ^{75}As (circles) and ^{71}Ga (squares) central transition after a CP-X (solid symbols) or CP-Y (empty symbols) sequence as a function of refocusing cycle number n.

nating pulse carrier phase discussed in the main text on the frequency offset δ. Solid lines correspond to sets of simulations with different π-pulse numbers. Thus for example, the orange line in Supplementary Fig. 3a shows the relative change of the z-component $M_z/M_z(0)$ of a magnetisation vector $\mathbf{M}$ (with $\mathbf{M}(0) = (0, 0, 1)^{\mathrm{T}}$) after evolution under a CP-X sequence $(\pi_x/2-(\pi_x-\pi_{-x})^8-\pi_x/2)$ for resonance frequency offsets $-1/(2T_{\mathrm{Rabi}}) \leq \delta \leq 1/(2T_{\mathrm{Rabi}})$. For comparison, experimental cw NMR spectra of the ^{71}Ga and ^{75}As central spin transitions rescaled by the typical respective experimental Rabi periods ($T_{\mathrm{Rabi}}(^{71}\mathrm{Ga}) = 5.6\ \mu$s and $T_{\mathrm{Rabi}}(^{75}\mathrm{As}) = 9.8\ \mu$s at rf power $P = 200$ W) are shown as dotted lines.

The desired performance is characterized by $M_z/M_z(0) \approx 1$ over a wide range of offsets δ. In this respect, we note that the CP-X cycle (Supplementary Fig. 3a) is very robust against frequency

offsets: even at large cycle numbers, the magnetisation vector is largely restored over a broad range of the offset frequencies. In practice, this means that we can expect a stable NMR echo amplitude at short evolution times independent of the number of applied π-pulses (as long as additional pulse calibration errors are negligible). This expected behaviour is shown in Supplementary Fig. 3c: here, we calculate the expected experimental echo amplitude from a weighted average of the normalised final magnetisation over the respective ^{71}Ga and ^{75}As NMR spectra for different cycle numbers n. In agreement with the experimental data shown in Fig. 3d-f of the main text, the CP-X signal amplitude (solid symbols) is stable. By contrast, we note from Supplementary Fig. 3b,c that the bandwidth within which the CP-Y sequence can restore the initial magnetisation rapidly narrows with increasing cycle number n. Again, this is in agreement with the experimental observation in

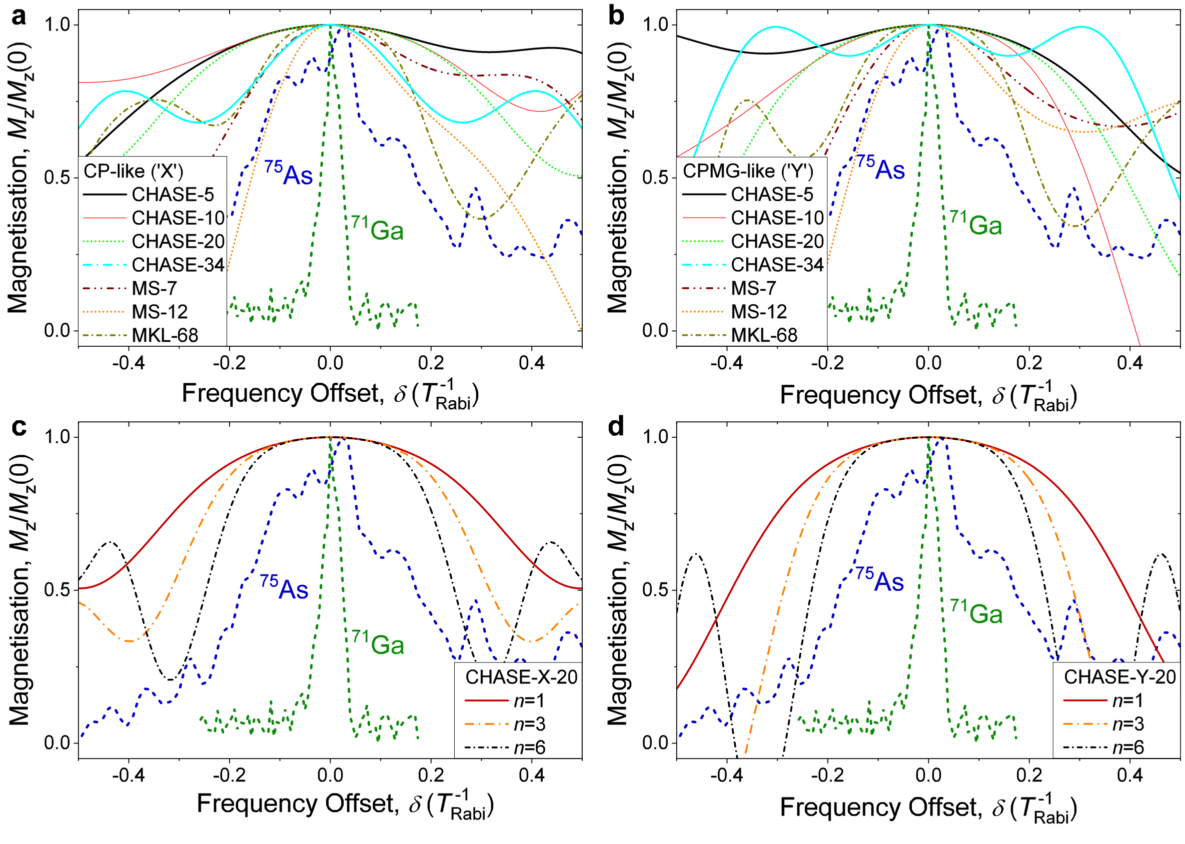

Supplementary Figure 4. **Analysis of the broadband refocusing performance of the CHASE, MS and MKL pulse sequences. a,b** Normalised magnetisation along the $\hat{e}_z$ axis after evolution under a series of non-resonant rf pulses as a function of the resonance frequency offset δ for (a) CHASE-X and (b) CHASE-Y sequences and additional cycles from literature. Dotted lines show normalised NMR spectra of the ^{75}As (blue) and ^{71}Ga (green) central transitions with frequency axes rescaled by T_{Rabi}. **c,d** Simulated data for CHASE-X/Y-20 with different cycle numbers n.

the main text, where the CP-Y NMR spin echo amplitude $\Delta E_{\text{hf}}(\tau_{\text{evol}} \to 0)$ rapidly decreases with increasing π-pulse number. Although periodic revivals of the restored magnetisation are seen in the orange line of Supplementary Fig. 3b, these are experimentally compensated by the contributions of spins at intermediate offsets δ where the final magnetisation vector is effectively flipped. This oscillating behaviour arises as the total pulse rotation error adds up to multiple precessions of the magnetisation vector $\mathbf{M}$ about $\hat{e}_\Omega$.

We run the same simulations for the CHASE sequences introduced in the main text and for the alternative sequences from literature analysed in Supplementary Note 1. The results for the various cycles under X- and Y- initialisation pulses are depicted in Supplementary Figs 4a and 4b, respectively. We note that most sequences have a similar 'hard pulse' bandwidth under both initialisation pulse conditions. Overall, all of the sequences presented are more stable against resonance offsets than the CP-Y sequence studied in the main text. However, the broadband performance of the CP-X sequence (Supplementary Fig. 3a) remains higher than that of any CHASE cycle. This is in qualitative agreement with our experimental results (compare Fig. 3d-f of the main text).

Additionally, we see that multiple cycles of CHASE-X/Y-20 reduce the offset tolerance to some extent (Supplementary Fig. 4c,d). This is confirmed experimentally, as the echo amplitude of the spectrally broader ^{75}As ensemble is noticeably reduced with increasing cycle number in Fig. 3e of the main text.

In summary, the reduced NMR echo amplitudes at short free evolution times observed in the experiments can be reproduced qualitatively using a simple Bloch model. We conclude that such echo amplitude reduction is not a fundamental limitation of the various pulse cycles we study, but can be attributed to 'soft' rf pulses which for a given spectral broadening of the spin bath can in principle be avoided by using higher rf excitation powers. Alternatively, more advanced NMR techniques such as composite pulses could be implemented in future experiments to increase the 'hardness' of the applied pulses[16,17]. This effect needs to be distinguished from the reduction of the spin coherence times T_2 observed in strongly inhomogeneous spin baths under multiple cycles of CHASE, which occurs even under ideal (infinitely fast) rotations.

Supplementary Note 4. NUMERICAL SIMULATION OF THE NUCLEAR SPIN EVOLUTION UNDER PULSED RADIOFREQUENCY MANIPULATION

In this Note we describe the details of the numerical simulation of the quantum mechanical evolution dynamics of the interacting nuclear spin bath.

The model

We consider once again the model introduced in Supplementary Note 1, where the evolution of the wavefunction $\psi(t)$ describing the state of the nuclear spin bath in the rotating frame of an external magnetic field B_{z} is determined by:

$$\begin{aligned} \partial\psi(t)/\partial t &= -(\mathrm{i}/\hbar)\mathcal{H}(t)\psi(t)\ , \\ \mathcal{H}(t) &= \mathcal{H}_0^{\mathrm{z}} + \mathcal{H}_{\mathrm{d}}^{\mathrm{zz}} + \mathcal{H}_{\mathrm{rf}}(t)\ . \end{aligned} \tag{22}$$

As before, the Hamiltonian $\mathcal{H}(t)$ is composed of a term $\mathcal{H}_0^{\mathrm{z}}$ describing here quadrupolar interaction with electric field gradients, a nuclear dipolar coupling term $\mathcal{H}_{\mathrm{d}}^{\mathrm{zz}}$ and a radio-frequency (rf) term $\mathcal{H}_{\mathrm{rf}}(t)$.

We consider half-integer spins I and simulate evolution only of the $I_{\mathrm{z}} = \pm 1/2$ subspace corresponding to the NMR experiments on the central transition. The effect of the quadrupolar interaction (more specifically of its second order term) on the $I_{\mathrm{z}} = \pm 1/2$ manifold is equivalent to an additional magnetic field that changes the Larmor frequency of the i-th nucleus by $\Delta\nu_i$. The quadrupolar term can then be written explicitly as:

$$\mathcal{H}_0^{\mathrm{z}} = 2\pi\hbar\sum_{i=1}^{N}\Delta\nu_i I_{i,\mathrm{z}}\ , \tag{23}$$

where the summation goes over all N nuclei.

We consider the case of high magnetic field (significantly larger than the local dipolar field), so that the nuclear-nuclear interaction is described by the truncated dipole-dipole Hamiltonian:

$$\mathcal{H}_{\mathrm{d}}^{\mathrm{zz}} = \frac{\mu_0}{4\pi}\hbar^2\gamma^2\sum_{i<j}\frac{x_{i,j}^2 + y_{i,j}^2 - 2z_{i,j}^2}{(x_{i,j}^2 + y_{i,j}^2 + z_{i,j}^2)^{5/2}}\left[I_{i,\mathrm{z}}I_{j,\mathrm{z}} - \frac{(I+1/2)^2}{2}(I_{i,\mathrm{x}}I_{j,\mathrm{x}} + I_{i,\mathrm{y}}I_{j,\mathrm{y}})\right]\ , \tag{24}$$

where $\mu_0 = 4\pi \times 10^{-7}$ N A^{-2} is the magnetic constant, γ is the nuclear gyromagnetic ratio, $(x_{i,j}, y_{i,j}, z_{i,j})$ is the vector connecting spins i and j and the summation goes over all pairs of non-identical nuclei. We ignore here any possible contributions from pseudo-dipolar or exchange interactions.

The effect of the rf field is described by:

$$\mathcal{H}_{\mathrm{rf}}(t) = 2\pi\hbar(I+1/2)\sum_{i=1}^{N}\left(\nu_{1,\mathrm{x}}(t)I_{i,\mathrm{x}} + \nu_{1,\mathrm{y}}(t)I_{i,\mathrm{y}}\right) , \tag{25}$$

where parameters $\nu_{1,\mathrm{x}}(t)$ and $\nu_{1,\mathrm{y}}(t)$ characterise the amplitude of the rf magnetic field along the x and y axes of the rotating frame respectively. The parameters $\nu_{1,\mathrm{x}}(t)$ and $\nu_{1,\mathrm{y}}(t)$ are piecewise functions of time describing the variation of the rf field amplitude during the pulse sequence.

In the above equations (23), (24) and (25) the operators I_{x}, I_{y} and I_{z} are spin-1/2 Pauli matrices acting on the effective spin-1/2 subspace of the spin states with spin projections $I_{\mathrm{z}} = \pm 1/2$. The factors $(I + 1/2)$ in equations (24) and (25) [which differ them from equations (3), (4), (5)] arise from the matrix elements of the I_{x} and I_{y} operators of the full spin-I nuclei that are projected on to the $I_{\mathrm{z}} = \pm 1/2$ subspace. All quantities are in SI units, e.g. frequencies ν are in hertz and Hamiltonians are in joules.

The evolution of the nuclear spin magnetisation is calculated by direct propagation of the Schrödinger equation [Eq. (22)] using the 6-th order Runge-Kutta method implemented in Mathematica software versions 10.3, 11.0.1, or 11.3. The Hamiltonian matrices are stored and handled as sparse arrays for improved computation efficiency. For a given system of few spins, direct evaluation of Eq. (22) gives an exact evolution of the wavefunction, thus yielding a complete description of the nuclear spin dynamics. The maximum number of spins N that can be simulated with this direct approach is severely limited by the computational resources, since the memory and the computation time scale approximately as $\sim N^2$ and $\sim N^4$ respectively. Thus when performing simulations on a toy system with small N, the choice of the parameters and initial states becomes important for obtaining results that are relevant for systems with much larger N (such as quantum dots with $N \geq 10000$). The choice of parameters is discussed in the following section.

Model parameters for simulation of the nuclear spin dynamics in quantum dots

For simulations, nuclear spins are placed at the nodes of the face-centered cubic (fcc) lattice. One nucleus is placed at the origin $x = y = z = 0$ and the other nuclei are selected from its nearest neighbors – this way the nuclear spin cluster is kept as 'spherical' and 'dense' as possible which allows approximating the complexity and the magnitude of the dipolar interaction in a 3D lattice. Example clusters with $N = 6$, $N = 12$ and $N = 19$ spins are shown in Supplementary Fig. 5a. The nuclei are taken to be either ^{75}As with $I = 3/2$ and gyromagnetic ratio $\gamma = 2\pi \times 7.29 \times 10^6$ rad s^{-1}, or ^{115}In with $I = 9/2$ and gyromagnetic ratio $\gamma = 2\pi \times 9.38 \times 10^6$ rad s^{-1}. The lattice constant of

the fcc lattice is taken to be $a_0 = 0.564786$ nm, corresponding to GaAs at a temperature of $\sim$4 K.

Inhomogeneous quadrupolar interaction is introduced by varying the Larmor frequency $\nu_{\rm L}$ of each spin [Eq. (23)]. First, the Larmor frequency of the i-th spin $i \in [1, N]$ in the rotating frame is set to be $\Delta\nu_i = \nu_{\rm Q}(i - 1/2 - N/2)$. This equidistant set of $\Delta\nu_i$ is then rescaled as $\Delta\nu_i \rightarrow k_1 \times \Delta\nu_i$, preserving the mean Larmor frequency $\overline{\Delta\nu_i} = 0$. The factor k_1 is chosen as an implementation of a uniform random distribution in the range [0.77..1.3]. At the next step, each frequency $\Delta\nu_i$ is modified further by adding a random offset $k_{2,i} \times \nu_{\rm Q}$ with $k_{2,i}$ selected randomly for each nucleus from a uniform distribution in the range $[-0.325, 0.325]$. The purpose of such a randomisation using parameters k_1 and $k_{2,i}$ is to eliminate spurious periodic beatings in the nuclear spin dynamics arising from the small number of the nuclear spins $N < 20$ used in the simulations. Such beatings are not present in experimental decay curves on real III-V quantum dots where $N > 10000$, but similar features are observed for defect spins in dilute nuclear spin baths (e.g. NV centres in diamond), where electron-nuclear hyperfine coupling leads to periodic coherence collapses[18,19]. The physical meaning of the randomisation procedure can be seen as follows: The quantum dot can be viewed as built of a large number of clusters, each containing N spins with a different random distribution of quadrupolar frequencies. The experimentally measured NMR signal is an average over all such clusters, which is simulated by Monte-Carlo averaging over k_1 and $k_{2,i}$ in the numerical calculations. The step $\nu_{\rm Q}$ for the equidistant spacing of Larmor frequencies is chosen to be large compared to the dipolar coupling ν_{ij} of any two spins, so that the suppression of dipolar flip-flops arising from quadrupolar interaction (characteristic of self-assembled quantum dots[20]) can be efficiently simulated. We typically use $\nu_{\rm Q} = 2000$ Hz for ^{75}As and $\nu_{\rm Q} = 4000$ Hz for ^{115}In, which are chosen empirically by observing that no change in Hahn echo spin dynamics occurs when $\nu_{\rm Q}$ is increased further. The ranges for k_1 and $k_{2,i}$ are also chosen from trial simulations to be large enough to suppress spurious beatings while still small enough to ensure that the minimum difference between any $\Delta\nu_i$ is large enough to emulate strongly inhomogeneous quadrupolar interaction. When simulating spin dynamics of the nuclei in the absence of quadrupolar effects ($\Delta\nu_i \ll \nu_{ij}$) we use the above procedure with $\nu_{\rm Q}$, k_1 and $k_{2,i}$ set to zero.

For initialisation of the nuclear spin system we use the following procedure. Each of the N nuclear spins is randomly initialised in one of the four single-spin eigen states with $I_{\rm z} = -I .. + I$. The probabilities of finding each nucleus in $I_{\rm z} = \pm 1/2$ states and $|I_{\rm z}| > 1/2$ are taken to be 60% and 40% respectively. The probabilities for the $I_{\rm z} = +1/2$ and $I_{\rm z} = -1/2$ states are taken to produce 75% polarisation degree in the $I_{\rm z} = \pm 1/2$ subensemble. Such a choice of probabilities corresponds

closely to the experimental conditions where optical pumping inducing nuclear spin polarisation degree of ∼50% is followed by adiabatic radiofrequency sweeps exchanging the populations of $I_z = \pm 1/2$ and $I_z = \pm I$ states[20]. (Additional simulations show that initial polarization in the range 10% − 100% has little effect on spin echo decay times.) The nuclei in the $|I_z| > 1/2$ states are then ignored when simulating the spin dynamics of the $I_z = \pm 1/2$ states. This is justified since the $|I_z| > 1/2$ states have very long correlation times (τ_c ∼10 s, Ref.[21]) and act on the $I_z = \pm 1/2$ spins simply as a source of quasistatic local magnetic fields which are already taken into account by the inhomogeneous spread of the Larmor frequencies $\Delta\nu_i$. This initialisation procedure gives a tensor product random state which is not an eigenstate but where each nucleus is in a single-spin eigenstate with $I_z = +1/2$ or $I_z = -1/2$. In each simulation run (Monte-Carlo sample) the initial wavefunction is constructed by repeating the above procedure and creating a linear superposition of 1000 basic random states with random complex weighting coefficients. Such a highly entangled pure state with finite polarisation along z direction has self-averaging properties arising from quantum parallelism[22] and allows for faster convergence of the Monte-Carlo simulations.

The typical number of Monte-Carlo samples is 1000. For each Monte-Carlo sample a set of nuclear frequency shifts $\Delta\nu_i$ is generated (with random parameters k_1 and $k_{2,i}$), the wavefunction is then initialised into a random superposition state as described above. The time evolution of the wavefunction is then calculated numerically from the Schrödinger equation (22) with a Hamiltonian whose time dependence is a piecewise function determined by the rf pulse sequences. The overall time dependence of the nuclear spin polarisation is calculated by averaging over the Monte-Carlo samples. In all simulations the nuclei are initialised at $t = 0$ in a state polarised along the $\hat{e}_z$ axis before a single initialisation $\pi/2$-pulse is used to rotate the polarisation into the xy plane. Then a cyclic time sequence consisting of rf pulse rotations and free evolution periods is simulated. Finally a single $\pi/2$-pulse is used to rotate the magnetisation in the direction opposite to that of the initialisation pulse. All of the studied NMR pulse sequences are cyclic, i.e. in the limit of a short free evolution and ideal rf pulses the magnetisation is returned into its original state along the $\hat{e}_z$ axis (or into a state with inverted z-magnetisation for HE-Y, the Meiboom-Gill version of Hahn echo). In simulations we use both ideal (infinitely short, or 'hard') and non-ideal (finite duration, or 'soft') rectangular rf pulses. The total free evolution time τ_{evol} is varied, and for each value of τ_{evol} the final value of the nuclear magnetisation $\langle I_z \rangle$ along the $\hat{e}_z$ axis is computed. The resulting time dependence $\langle I_z(\tau_{evol}) \rangle$ reflects the process of nuclear spin echo decoherence and can be used to derive the coherence time T_2.

In the numerical simulations the nuclear spin wavefunction can be found explicitly at any

time t during the pulse sequence. This wavefunction can be used to evaluate not only the spin magnetisation components $I_{\mathrm{x}}(t)$, $I_{\mathrm{y}}(t)$, $I_{\mathrm{z}}(t)$ but also the degree of spin-spin entanglement $C_{\mathrm{I}}(t)$ at each time t. Entanglement of a quantum state can be quantified as a distance of this state to the set $\mathcal{D}$ of all disentangled states[23]. Here we follow Ref.[24] and quantify entanglement using intrinsic coherence C_{I} which relies on the quantum version of the Jensen-Shannon divergence (QJSD) as a distance measure. The states in $\mathcal{D}$ are taken to be statistical mixtures (with probabilities p_k) of the states that are products of single-spin basis states (Eq. 3 in Ref.[24]). We find the minimum C_{I} by varying p_k and the single-spin basis states (Eq. 4 in Ref.[24]). The number of products in the statistical mixture is limited to three ($k = 1..3$), which is found to give C_{I} with good accuracy within a reasonable computation time. For entanglement evolution calculations we use the same settings as for the spin echo decay simulations, except that the total number of spins is $N = 9$ with the number of spins in the $I_{\mathrm{z}} = \pm 1/2$ states restricted between 4 and 7 to reduce the computation time, and the initial state is chosen to be one of the eigenstates of the spin system avoiding additional initial entanglement arising in a superposition state. In case of a large inhomogeneous broadening ($\Delta\nu_i \gg \nu_{ij}$) many-body spin eigenstates have low entanglement $C_{\mathrm{I}} \approx 0.02$ for ^{75}As, $C_{\mathrm{I}} \approx 0.07$ for ^{115}In, whereas $C_{\mathrm{I}} \approx 0.3$ in case of eigenstates under small inhomogeneous broadening ($\Delta\nu_i \ll \nu_{ij}$).

Examples of simulations of the nuclear spin dynamics in quantum dots: dependence on the number of spins N

We now give several examples of the results obtained from the above described numerical simulation procedure. Several simulated $\langle I_{\mathrm{z}}(\tau_{\mathrm{evol}})\rangle$ curves are shown in Supplementary Fig. 5b for a Meiboom-Gill version Hahn echo (HE-Y, solid symbols) and CHASE-Y-20 (open symbols) pulse sequences – these are computed for clusters with different numbers of nuclei shown in Supplementary Fig. 5a for the case of large inhomogeneous quadrupolar interaction ($\Delta\nu_i \gg \nu_{ij}$). Lines show fitting using compressed exponential functions $\langle I_{\mathrm{z}}(\tau_{\mathrm{evol}})\rangle = \langle I_{\mathrm{z}}(\tau_{\mathrm{evol}} \to 0)\rangle \cdot \mathrm{e}^{-(\tau_{\mathrm{evol}}/T_2)^{\beta}}$ (same as Eq. 3 of the main text). For the Hahn echo sequence the decay is close to Gaussian (characterised by compression factor $\beta \approx 2.0 - 2.1$), while for CHASE-Y-20 the best fit is for $\beta \approx 1.56 - 1.67$, and some deviation from a mono-exponential decay is observed, especially at small N.

The nuclear spin decoherence times T_2 derived from the fits as in Supplementary Fig. 5b are shown in Supplementary Fig. 5c by the symbols and are compared to the experimental values for ^{75}As spins in self-assembled quantum dots (shaded areas). It can be seen that the number of spins affects the overall timescale of the nuclear spin decoherence – for larger N the nuclear spin

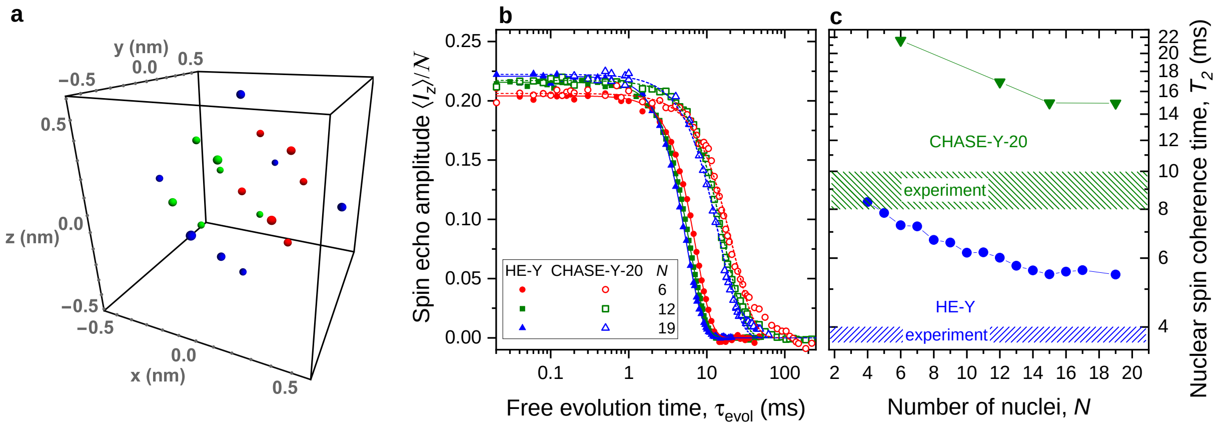

Supplementary Figure 5. **Model system and results of first-principle simulations of the nuclear spin dynamics under pulse control sequences.** **a** Spatial geometry of the ^{75}As nuclear spin cluster used for numerical simulations. Red balls show a configuration with N=6 spins, red and green with N=12 spins, red, green and blue combined together form a cluster with N=19 spins. **b** Simulated dependence of the NMR spin echo amplitude (i.e. nuclear spin magnetisation $\langle I_z \rangle$ at the end of spin evolution normalised by the number of spins N) on the total free evolution time $\tau_{\rm evol}$ under Hahn Echo (HE-Y, solid symbols) and CHASE-Y-20 (open symbols) pulse sequences computed for N=6 spins (circles), N=12 spins (squares), and N=19 spins (triangles). A Meiboom-Gill version of the Hahn echo sequence ($\pi/2_{\rm y} - \tau_{\rm evol}/2 - \pi_{\rm x} - \tau_{\rm evol}/2 - \pi/2_{-\rm y}$) is used with a $\pi/2$-shift between the rf carrier phases of the $\pi/2$- and π-pulses. Lines show the fitting used to derive nuclear spin decoherence times T_2. **c** Nuclear spin decoherence times T_2 derived from numerical simulations plotted as a function of the number of nuclear spins for Hahn Echo (circles) and CHASE-Y-20 (triangles) pulse sequences. Shaded areas show experimentally measured decoherence times of ^{75}As spins in a self-assembled quantum dot.

decoherence is faster as the interaction with a larger number of neighbors is taken into account. However, the overall trend in variation of T_2 under different pulse sequences is found to be robust against N. For example, while the decoherence times T_2 depend on N as shown in Fig. 5c, the ratio of the T_2 values under CHASE-Y-20 and Hahn echo sequences is nearly independent of N, ranging between ~ 2.74 for $N = 19$ and ~ 3.0 for $N = 6$ which is in good agreement with the experimental ratio of ~ 2.4. These test results justify the use of relatively small N – for most simulations in this work we employ $N = 12$ giving a good compromise between accuracy and computation time. The fact that the T_2 simulated for $N = 12$ differs from the experimental T_2 of a mesoscopic system with $N \sim 10000$ only by $\sim$50% indicates the robustness of our approach. Thus our simulations (i) give a good quantitative numerical estimate of the absolute T_2 values, and (ii) provide an excellent tool for examining the effect of various pulse sequences on T_2.

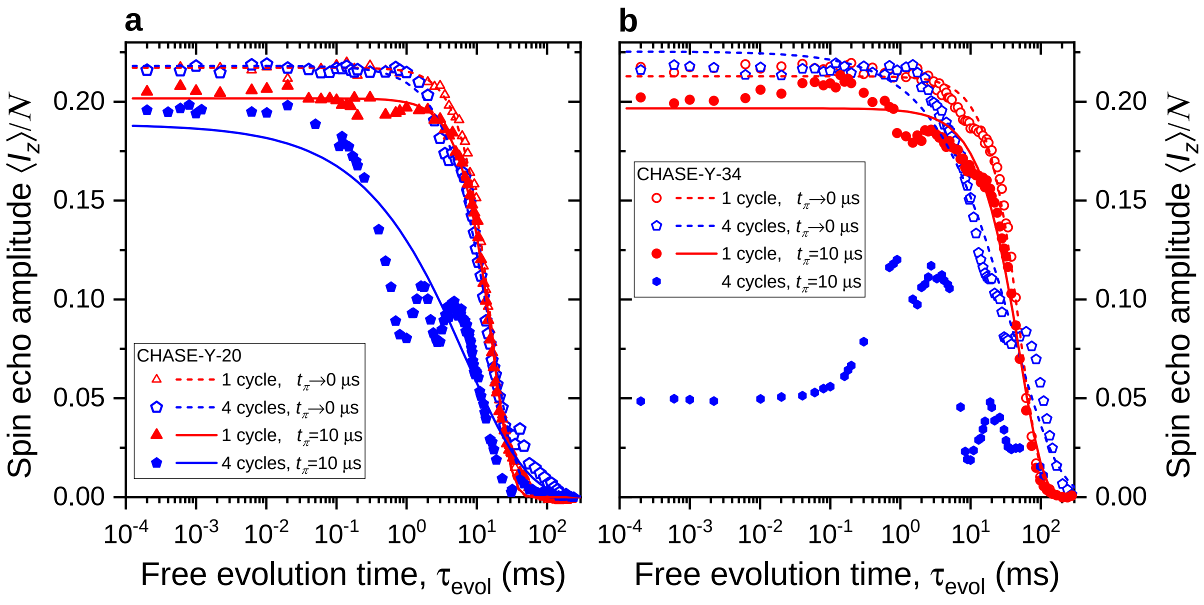

Supplementary Figure 6. **Examples of first-principle simulations results and derivation of the nuclear spin coherence times. a** Simulated dependence of the NMR spin echo amplitude (i.e. nuclear spin magnetisation $\langle I_z \rangle$ at the end of spin evolution normalised by the number of spins N) on the total free evolution time τ_{evol} under CHASE-Y-20 pulse sequence computed for N =12 spins. **b** Same for CHASE-Y-34. The results are presented for infinitely short ($t_\pi \to 0$, open symbols) and finite ($t_\pi = 10\ \mu$s, solid symbols) control pulses. The simulations were performed for 1 cycle (triangles) and for 4 cycles (pentagons) of the sequence. Lines show best least-squares fits using compressed exponents. In the case of 4 cycles of CHASE-Y-34 with $t_\pi = 10\ \mu$s the imperfect pulse rotations result in significant loss of transverse nuclear spin magnetisation even at short τ_{evol} – this prohibits unambiguous definition of the coherence time T_2, thus no fitting results are shown.

Procedure for derivation of the nuclear spin coherence times and echo amplitudes from the results of numerical simulations

We now present the raw data of the numerical simulations for the CHASE sequences (Supplementary Fig. 6) and discuss the procedure for analysing the raw data and deriving the spin bath coherence times T_2 and the echo amplitudes $\langle I_z(\tau_{\mathrm{evol}} \to 0)\rangle$ in the limit of short free evolution time $\tau_{\mathrm{evol}} \to 0$. Supplementary Fig. 6a shows the simulated spin bath dynamics under CHASE-Y-20. The results are presented for ideal infinitely short ('hard') rf control pulses (open symbols) and for the finite ('soft') rectangular pulses (solid symbols, π-pulse length of $t_\pi = 10\ \mu$s). The simulations were performed for 1 cycle of the sequence (triangles) and for 4 cycles (pentagons). Lines show best least-squares fits using compressed exponents (Eq. 3 of the main text). These fits are used to derive

the spin bath coherence times T_2. While for 1 cycle the fit is good, for more complex conditions, e.g. 4 cycles and $t_\pi = 10$ μs, there is a considerable deviation between numerical simulations and exponential fits – in such cases the T_2 times are still derived from fitting but should be treated as approximate values.

Supplementary Fig. 6b shows further results for the spin bath dynamics under the CHASE-Y-34 pulse sequence. Here deviation from the exponential fit is observed for 4 cycles even at $t_\pi \to 0$ while at $t_\pi = 10$ μs the oscillations and reduction of the echo amplitude at short free evolution time $\tau_{\mathrm{evol}} \to 0$ are particularly pronounced. In case of spin-9/2 nuclei we find even stronger deviations from a mono-exponential echo decay, with signatures of a two-stage decay. This requires care when deriving decoherence parameters. Firstly, in our analysis the echo amplitude $\langle I_z(\tau_{\mathrm{evol}} \to 0)\rangle$ is derived not from fitting but rather by taking the average spin magnetisation $\langle I_z\rangle$ (normalised by the number of nuclei N) at short free evolution times $\tau_{\mathrm{evol}} < 5$ μs – this definition of $\langle I_z(\tau_{\mathrm{evol}} \to 0)\rangle$ is not affected by deviation of the spin decay from the exponential model. Secondly, for any cyclic pulse sequences with ideal 'hard' pulses ($t_\pi \to 0$) the resulting magnetisation $\langle I_z(\tau_{\mathrm{evol}} \to 0)\rangle$ after the sequence with short free evolution $\tau_{\mathrm{evol}} \to 0$ is by definition equal to the initial magnetisation $\langle I_z(t = 0)\rangle$ before the pulse sequence is applied (in the studied example $\langle I_z(t = 0)\rangle/N \approx 0.217$), while for non-ideal pulses ($t_\pi > 0$) nuclear spin magnetisation at $\tau_{\mathrm{evol}} \to 0$ may be lost simply due to the imperfect spin rotations (i.e. due to the 'soft' pulse conditions). Such imperfect rotations mean that the spin bath states during free evolution periods of finite duration $\tau_{\mathrm{evol}} > 0$ deviate from the desired sequence. Under such conditions (e.g. $t_\pi = 10$ μs in Supplementary Fig. 6) the reduction in $\langle I_z(\tau_{\mathrm{evol}})\rangle$ is not related to decoherence as such, prohibiting any unambiguous definition for T_2.

Taking into account the above arguments we establish an approach to the analysis of the numerical results which can be summarised as follows: The echo amplitude $\langle I_z(\tau_{\mathrm{evol}} \to 0)\rangle$ is derived by averaging the I_z over short free evolution times $\tau_{\mathrm{evol}} < 5$ μs. For echo amplitudes $\langle I_z(\tau_{\mathrm{evol}} \to 0)\rangle$ below 70% of the initial magnetisation $\langle I_z(t = 0)\rangle$ the coherence time T_2 is undefined, while for $\langle I_z(\tau_{\mathrm{evol}} \to 0)\rangle$ above this threshold, the T_2 is derived from fitting with compressed exponential functions. Moreover, in the main text and the subsequent discussion we present echo amplitudes at short free evolution times $\langle I_z(\tau_{\mathrm{evol}} \to 0)\rangle$ normalised by the initial magnetisation $\langle I_z(t = 0)\rangle$.

Supplementary Note 5. ADDITIONAL RESULTS OF NUMERICAL SIMULATIONS OF THE NUCLEAR SPIN EVOLUTION UNDER DIFFERENT PULSE SEQUENCES

In the main text we present the results of numerical simulations for the pulse sequences used in the experimental work. Simulations are in good agreement with the experiment and confirm robust extension of the nuclear spin coherence time T_2 under CHASE pulse sequences. In this section we present simulated nuclear spin dynamics under alternative pulse sequences reported in the literature and compare their performance to CHASE.

Having discussed in Supplementary Note 4 how echo amplitudes $\langle I_z(\tau_{evol} \to 0)\rangle$ and coherence times T_2 are derived, we now examine their dependence on the control pulse sequence parameters. The results of the simulations are summarised in Supplementary Fig. 7 for the cases of small inhomogeneous quadrupolar interaction ($\Delta\nu_i \ll \nu_{ij}$, panels a, c) and large inhomogeneous quadrupolar interaction ($\Delta\nu_i \gg \nu_{ij}$, panels b, d). Four types of sequences are presented: (i) CHASE-10/20 and CHASE-34 as proposed in this work, (ii) the 7 and 12 pulse sequences proposed theoretically by Moiseev and Skrebnev[9,10] and labeled MS-7 and MS-12 here, (iii) the sequence consisting of 8 MREV-8 pulse trains interwoven with four phase-refocusing π-pulses used by Maurer, Kucsko et al.[11] in experiments on NV centers in diamond and labeled MKL-68, (iv) the 'time-suspension' sequence consisting of 48 $\pi/2$-pulses as introduced by Cory et al.[25] (labeled CMG-48). The results for Hahn Echo (HE) and Carr-Parcell (CP) sequences are shown as well for a reference. The numbers in the sequence labels stand for the total number of rf control pulses in one cycle. All results in Supplementary Fig. 7 are plotted as a function of the total duration of the control rf pulses (total gate time) in the units of the π-pulse duration t_π. Similar to the way the results are presented in the main text, we combine CHASE-10 with CHASE-20 and MS-7 with MS-12: the points with the shortest total gate time correspond to one cycle of CHASE-10 and MS-7 sequences, while points with larger gate times correspond to integer numbers of repeated cycles of CHASE-20 and MS-12. For each sequence we consider two cases: with nuclear magnetisation initialised by a $\pi/2$-pulse along the same $\hat{e}_x$ axis as the π-pulses of the sequence (-X sequences) and with initialisation along the $\hat{e}_y$ axis, orthogonal to that of the π pulses (Meiboom-Gill version, labeled -Y).

The case of negligible inhomogeneous resonance broadening

We first examine the case of small inhomogeneous resonance broadening $\Delta\nu_i \ll \nu_{ij}$ (negligible quadrupolar effects or chemical shifts) as shown in Supplementary Fig. 7a,c. It follows from

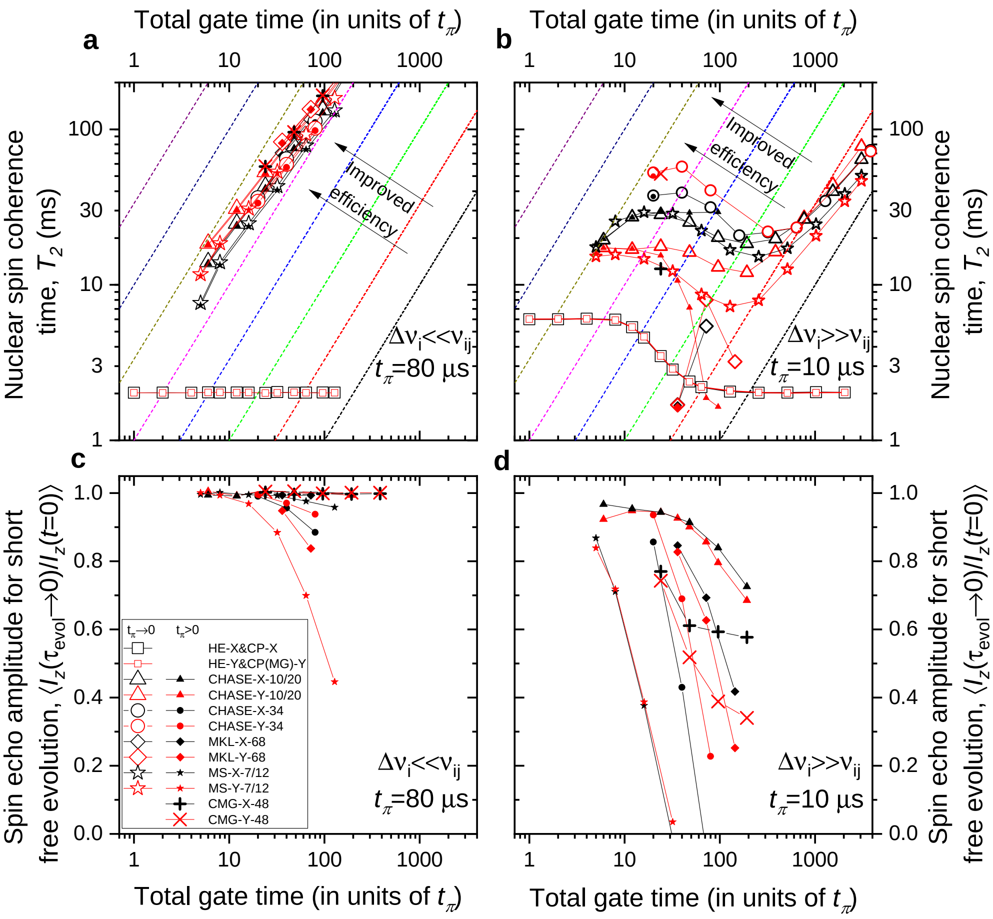

Supplementary Figure 7. **Nuclear spin coherence times and echo amplitudes under rf pulse control sequences derived from numerical simulations on $N = 12$ dipolar coupled ^{75}As nuclear spins.** **a,c** Results for the case of small $\Delta\nu_i \ll \Delta_{ij}$ inhomogeneous (quadrupolar) broadening. **b,d** Same for the case of large $\Delta\nu_i \gg \Delta_{ij}$ inhomogeneous broadening. Symbols in panels (a) and (b) show the nuclear spin coherence times T_2 for different pulse sequences as a function of the total pulse (gate) time in units of t_π. The plot for each type of sequence is obtained by varying the number of cycle repeats; for Hahn Echo (HE), MS-7 and CHASE-10 we consider only one cycle and combine the data with CP, MS-12 and CHASE-20 respectively. The dashed lines represent constant efficiencies of the pulse sequences, defined as coherence time to gate time ratio. The gate time dependencies of the NMR spin echo amplitude (final magnetisation) at short free evolution $\langle I_z(\tau_{evol} \to 0)\rangle$ are shown in (c) and (d), the values are normalised by the magnitude of the initial magnetisation $\langle I_z(t = 0)\rangle$. Simulations are carried out for both infinitely short ($t_\pi \to 0$, open symbols) and finite pulses (solid symbols), where we set $t_\pi = 80$ μs for $\Delta\nu_i \ll \nu_{ij}$ and $t_\pi = 10$ μs for $\Delta\nu_i \gg \nu_{ij}$. The $\langle I_z(\tau_{evol} \to 0)\rangle$ values in (c) and (d) are plotted only for $t_\pi > 0$ since at $t_\pi \to 0$ one has $\langle I_z(\tau_{evol} \to 0)\rangle/\langle I_z(t = 0)\rangle = 1$ for any cyclic control pulse sequence by definition.

Supplementary Fig. 7a that all four types of sequences can be used to achieve arbitrarily long

nuclear spin coherence time: the T_2 increases approximately linearly with the increasing number of sequence repetitions (increasing total gate time). This is largely expected from AHT – when the number of cycles is increased, the cycle duration t_c is reduced, and the average Hamiltonian converges to its remaining zeroth order term $\bar{\mathcal{H}}_{\rm d}^{(0)}$. As shown in Supplementary Tables 1 and 2, this term vanishes for $t_\pi \rightarrow 0$ for all studied sequences. For a given total gate time, the T_2 values are very close for all four types of sequences for initial magnetisation along either $\hat{e}_{\rm x}$ or $\hat{e}_{\rm y}$ axes – the difference is less than a factor of 2. However, the performance of the sequences is notably different when non-ideal pulses ($t_\pi > 0$) are considered. For both MKL and MS sequences a pronounced loss of magnetisation $\langle I_{\rm z}(\tau_{\rm evol} \rightarrow 0)\rangle$ at short free evolution (echo amplitudes) is observed for the Meiboom-Gill (Y) versions of the sequences when the number of cycles is increased (Supplementary Fig. 7c) – this means that strong nuclear spin decoherence is induced by the finite 'soft' control pulses irrespective of the decoherence during free evolution between the pulses. By contrast the CHASE sequences show robust performance for an arbitrary direction of the initial nuclear spin magnetisation for the total gate times of up to $\sim 200 t_\pi$ studied here, thus demonstrating their capability to dynamically freeze arbitrary fluctuation of the transverse nuclear magnetisation. Similarly good performance under finite pulses is observed only for the CMG-48 'time-suspension' sequence[25] (crosses in Supplementary Fig. 7c).

The case of large inhomogeneous resonance broadening

The case of large inhomogeneous resonance broadening $\Delta\nu_i \gg \nu_{ij}$ (e.g. strong quadrupolar effects) is presented in Supplementary Fig. 7b,d. We start by examining the coherence times under ideal 'hard' control pulses ($t_\pi \rightarrow 0$, open symbols in Supplementary Fig. 7b). The MKL sequence exhibits reduced T_2 times, which are even shorter (for 1 cycle) than in the case of simple π-pulse trains (Carr-Parcell sequences, CP). This is likely due to the fact that the MKL sequence was not designed to be applied to strongly inhomogeneous spin systems in the first place. By contrast, all of the CHASE and MS sequences provide enhancement in T_2 compared to Hahn echo and CP and show a similar non-monotonic behaviour on the total gate time which is also presented in Fig. 4e,f of the main text for CHASE-10/20 sequences. For the total gate times up to $\sim 100 t_\pi - 200 t_\pi$ the nuclear spin coherence time T_2 is seen to decrease. Such reduction is also observed for the CP sequences and is interpreted to arise from fast rotations of the spins by the rf pulses which lead to an effectively shortened spin lifetime and broadened nuclear spin transitions. Such a broadening can compensate for the energy mismatch between the spins induced by the quadrupolar inhomogeneity

and restores the dipolar exchange spin-spin flip-flops. This interpretation is readily confirmed by examining the CP results: for a large number of pulse cycles (with the total gate time $\gtrsim 100t_\pi$) the T_2 of the inhomogeneous ($\Delta\nu_i \gg \nu_{ij}$, Supplementary Fig. 7b) nuclear spin bath reduces to exactly the value of $T_2 \approx 2.02$ ms observed for the homogeneous bath ($\Delta\nu_i \ll \nu_{ij}$, Supplementary Fig. 7a) where dipolar flip-flops are allowed. When the number of CHASE or MS sequence cycles is increased further ($\gtrsim 500t_\pi$ in Supplementary Fig. 7b), T_2 increases steadily, indicating suppression of dipolar interactions and convergence of the average Hamiltonian to zero, similar to the homogeneous case ($\Delta\nu_i \ll \nu_{ij}$, Supplementary Fig. 7a). The interplay between the opposing effects of the reappearance of the flip-flops and the convergence of the average Hamiltonian depends strongly on the magnitude of the quadrupolar inhomogeneity and rf pulse duration t_π. However, it is possible to establish a qualitative agreement between the experiment and the simulations: for a wide range of the CHASE-10/20 cycle numbers ($\lesssim 200t_\pi$ in Supplementary Fig. 7b), T_2 is nearly constant – this matches the weak dependence of the experimentally measured T_2 on the number of cycles as observed in Fig. 3b of the main text.

We now examine the effect of the finite 'soft' pulses ($t_\pi > 0$) under strong inhomogeneous broadening conditions ($\Delta\nu_i \gg \nu_{ij}$, solid symbols in Supplementary Fig. 7b,d). It follows from Supplementary Fig. 7d that the loss of transverse spin polarisation during the control rf pulses (observed as decrease in the initial echo amplitude $\langle I_z(\tau_{\mathrm{evol}} \to 0)\rangle$) is most pronounced for the MS sequences – the spin coherence can be maintained well above $\langle I_z(\tau_{\mathrm{evol}} \to 0)\rangle \sim 0.7$ only for one cycle of MS-7. One cycle of MKL-68 with a total gate time of $36t_\pi$ can preserve the echo amplitude above the 70% threshold but the resulting coherence time $T_2 < 2$ ms is shorter than for Hahn echo. Similarly, there is a strong loss of echo amplitude even for one cycle of the 'time-suspension' sequence[25] CMG-48 (crosses in Supplementary Fig. 7d, only finite-pulse results are shown since ideal pulses $t_\pi \to 0$ yield even shorter T_2 values than under $t_\pi > 0$). By contrast, the CHASE sequences demonstrate the best performance in terms of both preserving the echo amplitude $\langle I_z(\tau_{\mathrm{evol}} \to 0)\rangle$ under long rf pulse trains and enhancing the coherence time T_2. While CHASE-20 can maintain $\langle I_z(\tau_{\mathrm{evol}} \to 0)\rangle > 0.7$ for gate times $> 100t_\pi$, the coherence time T_2 decreases abruptly above $24t_\pi$ in case of the Y- initialisation pulse. A robust performance in terms of freezing of the spin bath fluctuation using finite pulses is obtained for either CHASE-10/20 or CHASE-34 for the total rf pulse gate times up to $20t_\pi - 24t_\pi$, with CHASE-34 producing a longer coherence time T_2.

Analysis and discussion

In various applications of magnetic resonance it is a common aim to seek for an optimal shape of the rf control field that produces the desired spin manipulation[26,27]. It is thus useful to compare the different pulse sequences discussed here by introducing a quantity that characterises their efficiency. To this end we take the ratio of the coherence time T_2 during free evolution and the duration of the rf control pulses required to achieve such T_2 – in other words, the pulse sequence is considered to be most efficient if it yields the largest increase in T_2 at a smallest possible overhead of spin manipulation via the rf control pulses. The dashed lines in Supplementary Fig. 7a,b show constant efficiency levels (given by linear functions with different slopes). It follows from Supplementary Fig. 7a that in case of negligible inhomogeneous resonance broadening ($\Delta\nu_i \ll \nu_{ij}$) the efficiency is nearly invariant, gradually decreasing with the growing number of sequence cycle repeats. In case of large inhomogeneity ($\Delta\nu_i \gg \nu_{ij}$) the increase in the number of sequence cycle repeats (total gate time) leads to reduction in efficiency due to the re-appearance of the dipolar flip-flops discussed above. Supplementary Fig. 7b shows that the best efficiency is achieved for one cycle of either MS-7 or CHASE-10, while for one cycle of CHASE-34 the coherence time T_2 can be extended only with some loss in efficiency. These results indicate that when the dipolar-coupled spin bath is inhomogeneously broadened (Supplementary Fig. 7b) its coherence can be extended efficiently only by introducing complex pulse sequences that cancel higher order terms of the averaged spin Hamiltonian – this is different from the case of negligible inhomogeneous broadening (Supplementary Fig. 7a) where cycles of the basic sequence repeated multiple times efficiently enhance the spin bath coherence.

Further improvements in simultaneous suppression of inhomogeneous spectral broadening and dipolar couplings in nuclear spin baths may be achieved by benchmarking the performance of the pulse sequences using techniques beyond AHT. Optimized control pulses beyond simple rectangular pulses used here may offer further improvements. One example of such a technique are composite pulses. We have conducted preliminary numerical simulations with modified CHASE sequences, where each pulse is replaced by a composite broadband BB1 pulse[28]. However, these pulses give no improvement and in fact result in a slight reduction of the nuclear spin coherence times T_2, while requiring significantly longer gate times (and hence reduced efficiency). Alternative approaches may involve more sophisticated tools, including numerical optimization algorithms[29].

To summarise the results of these numerical simulations, we find that the most efficient control of the spin bath coherence is achieved using one cycle of the CHASE-10, CHASE-20, or CHASE-

34 sequences as they (i) extend the spin bath coherence time T_2 both under small and large inhomogeneous resonance broadening, (ii) show robust preservation of the spin bath magnetisation even under non-ideal finite duration ('soft') control pulses, and (iii) effectively freeze nuclear spin fluctuations regardless of their direction in the plane perpendicular to the static external magnetic field. The simulations for CHASE-34 predict significant improvement of the coherence compared to CHASE-10/20 when applied to an inhomogeneously broadened system – this is confirmed in experiments on ^{75}As nuclear spins in self-assembled quantum dots. Overall, the CHASE sequences developed here provide a well balanced performance and can be used to control spin-1/2 and spin-3/2 fluctuations both in systems with large inhomogeneous resonance broadening (e.g. quantum dots) and systems with small broadening (e.g. defect spins in diamond) where good tolerance to non-ideal finite pulses is required. By contrast, as discussed in the main text, controlling the coherence of the spin-9/2 nuclei is inherently more challenging due to the strong flip-flop spin-spin coupling [Eq. (24)].

SUPPLEMENTARY REFERENCES

---

* andreas.waeber@tum.de

† e.chekhovich@sheffield.ac.uk

1 Haeberlen, U. and Waugh, J. S. Coherent Averaging Effects in Magnetic Resonance. *Physical Review* **175**, 453 (1968).

2 Rhim, W. K., Elleman, D. D., and Vaughan, R. W. Analysis of multiple pulse NMR in solids. *Journal of Chemical Physics* **59**, 3740 (1973).

3 Burum, D. P. and Rhim, W. K. Analysis of multiple pulse NMR in solids. III. *Journal of Chemical Physics* **71**, 944 (1979).

4 Slichter, C. P. *Principles of Magnetic Resonance*. Springer, corrected third edition, (1996).

5 Rhim, W. K., Elleman, D. D., and Vaughan, R. W. Enhanced resolution for solid state NMR. *Journal of Chemical Physics* **58**, 1772 (1973).

6 Mansfield, P., Orchard, M. J., Stalker, D. C., and Richards, K. H. B. Symmetrized Multipulse Nuclear-Magnetic-Resonance Experiments in Solids: Measurement of the Chemical-Shift Shielding Tensor in Some Compounds. *Physical Review B* **7**, 90 (1973).

7 Waugh, J. S., Huber, L. M., and Haeberlen, U. Approach to High-Resolution NMR in Solids. *Physical Review Letters* **20**, 180 (1968).

8 Helton, J. W. and de Oliveira, M. C. NCAlgebra. `http://math.ucsd.edu/~ncalg/`, (2017).

[9] Moiseev, S. A. and Skrebnev, V. A. Short-cycle pulse sequence for dynamical decoupling of local fields and dipole-dipole interactions. *Physical Review A* **91**, 022329 (2015).

[10] Moiseev, S. A. and Skrebnev, V. A. Symmetric-cycle pulse sequence for dynamical decoupling of local fields and dipole-dipole interactions. *Journal of Physics B* **48**, 135503 (2015).

[11] Maurer, P. C., Kucsko, G., Latta, C., Jiang, L., Yao, N. Y., Bennett, S. D., Pastawski, F., Hunger, D., Chisholm, N., Markham, M., Twitchen, D. J., Cirac, J. I., and Lukin, M. D. Room-Temperature Quantum Bit Memory Exceeding One Second. *Science* **336**, 1283 (2012).

[12] Li, D., Dong, Y., Ramos, R. G., Murray, J. D., MacLean, K., Dementyev, A. E., and Barrett, S. E. Intrinsic origin of spin echoes in dipolar solids generated by strong π pulses. *Physical Review B* **77**, 214306 (2008).

[13] Franzoni, M. B. and Levstein, P. R. Manifestations of the absence of spin diffusion in multipulse NMR experiments on diluted dipolar solids. *Physical Review B* **72**, 235410 (2005).

[14] Ridge, C. D., O'Donnell, L. F., and Walls, J. D. Long-lived selective spin echoes in dipolar solids under periodic and aperiodic π-pulse trains. *Physical Review B* **89**, 024404 (2014).

[15] Chekhovich, E. A., Kavokin, K. V., Puebla, J., Krysa, A. B., Hopkinson, M., Andreev, A. D., Sanchez, A. M., Beanland, R., Skolnick, M. S., and Tartakovskii, A. I. Structural analysis of strained quantum dots using nuclear magnetic resonance. *Nature Nanotechnology* **7**, 646 (2012).

[16] Levitt, M. H. and Freeman, R. NMR population inversion using a composite pulse. *Journal of Magnetic Resonance* **33**, 473 (1979).

[17] Tycko, R. Broadband population inversion. *Physical Review Letters* **51**, 775 (1983).

[18] Mims, W. B. Envelope Modulation in Spin-Echo Experiments. *Physical Review B* **5**, 2409 (1972).

[19] Jelezko, F., Gaebel, T., Popa, I., Gruber, A., and Wrachtrup, J. Observation of Coherent Oscillations in a Single Electron Spin. *Physical Review Letters* **92**, 076401 (2004).

[20] Chekhovich, E. A., Hopkinson, M., Skolnick, M. S., and Tartakovskii, A. I. Suppression of nuclear spin bath fluctuations in self-assembled quantum dots induced by inhomogeneous strain. *Nature Communications* **6**, 6348 (2015).

[21] Waeber, A. M., Hopkinson, M., Farrer, I., Ritchie, D. A., Nilsson, J., Stevenson, R. M., Bennett, A. J., Shields, A. J., Burkard, G., Tartakovskii, A. I., Skolnick, M. S., and Chekhovich, E. A. Few-second-long correlation times in a quantum dot nuclear spin bath probed by frequency-comb nuclear magnetic resonance spectroscopy. *Nature Physics* **12**, 688 (2016).

[22] Schliemann, J., Khaetskii, A. V., and Loss, D. Spin decay and quantum parallelism. *Physical Review B* **66**, 245303 (2002).

[23] Vedral, V., Plenio, M. B., Rippin, M. A., and Knight, P. L. Quantifying entanglement. *Phys. Rev. Lett.* **78**, 2275–2279 (1997).

[24] Radhakrishnan, C., Parthasarathy, M., Jambulingam, S., and Byrnes, T. Distribution of quantum coherence in multipartite systems. *Phys. Rev. Lett.* **116**, 150504 (2016).

[25] Cory, D. G., Miller, J. B., and Garroway, A. N. Time-suspension multiple-pulse sequences: applications

to solid-state imaging. *Journal of Magnetic Resonance* **90**, 205 – 213 (1990).

[26] Khaneja, N., Brockett, R., and Glaser, S. J. Time optimal control in spin systems. *Physical Review. A* **63**, 032308 (2001).

[27] Souza, A. M., Álvarez, G. A., and Suter, D. Robust Dynamical Decoupling for Quantum Computing and Quantum Memory. *Physical Review Letters* **106**, 240501 (2011).

[28] Wimperis, S. Broadband, Narrowband, and Passband Composite Pulses for Use in Advanced NMR Experiments. *Journal of Magnetic Resonance A* **109**, 221 (1994).

[29] Khaneja, N., Reiss, T., Kehlet, C., Schulte-Herbrüggen, T., and Glaser, S. J. Optimal control of coupled spin dynamics: design of NMR pulse sequences by gradient ascent algorithms. *Journal of Magnetic Resonance* **172**, 296 (2005).
